# BOD1 regulates the cerebellar IV/V lobe-fastigial nucleus circuit associated with motor coordination

**DOI:** 10.1038/s41392-022-00989-x

**Published:** 2022-06-01

**Authors:** Xiu-Xiu Liu, Xing-Hui Chen, Zhi-Wei Zheng, Qin Jiang, Chen Li, Lin Yang, Xiang Chen, Xing-Feng Mao, Hao-Yang Yuan, Li-Li Feng, Quan Jiang, Wei-Xing Shi, Takuya Sasaki, Kohji Fukunaga, Zhong Chen, Feng Han, Ying-Mei Lu

**Affiliations:** 1 Key Laboratory of Cardiovascular & Cerebrovascular Medicine, Drug Target and Drug Discovery Center, School of Pharmacy, Nanjing Medical University, 211166 Nanjing, China; 2 Department of Physiology, School of Basic Medical Sciences, Nanjing Medical University, 211166 Nanjing, China; 3 Institute of Pharmacology and Toxicology, College of Pharmaceutical Sciences, Zhejiang University, 310058 Hangzhou, China; 4 Department of Pharmaceutical and Administrative Sciences, Loma Linda University School of Pharmacy, Loma Linda, CA 92350 USA; 5 Department of Basic Sciences, Loma Linda University School of Medicine, Loma Linda, CA 92350 USA; 6 Department of Pharmacology, Graduate School of Pharmaceutical Sciences, Tohoku University, Sendai, 980-8578 Japan; 7 Institute of Brain Science, the Affiliated Brain Hospital of Nanjing Medical University, 210029 Nanjing, China; 8 Gusu School, Nanjing Medical University, Suzhou Municipal Hospital, The Affiliated Suzhou Hospital of Nanjing Medical University, 215002 Suzhou, China

**Keywords:** Diseases of the nervous system, Neurodevelopmental disorders

## Abstract

Cerebellar ataxias are characterized by a progressive decline in motor coordination, but the specific output circuits and underlying pathological mechanism remain poorly understood. Through cell-type-specific manipulations, we discovered a novel GABAergic Purkinje cell (PC) circuit in the cerebellar IV/V lobe that projected to CaMKIIα^+^ neurons in the fastigial nucleus (FN), which regulated sensorimotor coordination. Furthermore, transcriptomics profiling analysis revealed various cerebellar neuronal identities, and we validated that biorientation defective 1 (BOD1) played an important role in the circuit of IV/V lobe to FN. *BOD1* deficit in PCs of IV/V lobe attenuated the excitability and spine density of PCs, accompany with ataxia behaviors. Instead, BOD1 enrichment in PCs of IV/V lobe reversed the hyperexcitability of CaMKIIα^+^ neurons in the FN and ameliorated ataxia behaviors in *L7-Cre*; *BOD1*^*f/f*^ mice. Together, these findings further suggest that specific regulation of the cerebellar IV/V lobe^PCs ^→ FN^CaMKIIα+^ circuit might provide neuromodulatory targets for the treatment of ataxia behaviors.

## Introduction

Cerebellum dysfunction plays a pathogenic role in ataxias which are characterized by a progressive decline in motor coordination and eventual immobility.^1^ Purkinje cells (PCs) of the cerebellar cortex are an intrinsic factor in the neural mechanism underlying motor coordination.^2^ PCs are the most vulnerable neurons in the cerebellum, and their prominent loss is the main feature in the late phase of cerebellar neurodegenerative diseases.^3^ Inhibited or damaged PCs have been reported to cause a wide-based unsteady gait, extremity trajectory errors and poor accuracy performances in both animal models and human patients.^4,5^ Nevertheless, it must be noted that although related evidence demonstrates that PCs play a key role in motor coordination, the neural circuit mechanism by which PCs precisely regulate motor coordination remains unclarified.

Cerebellar cortex PCs send a strong projections to deep cerebellar (DCN).^6,7^ Tonic inhibition of PCs induces potent baseline inhibition of the DCN downstream target neurons, in which microcircuits may play an important role in maintaining motor pathway coordination.^8^ The cerebellar gene ablation or mutation reveals PCs have a significant role in ataxic phenotypes, cognitive disorders, and autistic-like behavior.^8,9^ More recently, Miterko and colleagues suggested that fully elucidating the PC properties is critical for further optimizing the clinical therapeutic strategy for ataxia.^10^ Therefore, profound changes in PC neurophysiology may lead to the cerebellar circuit dysfunction that explains the behavioral characteristics of patients with ataxia. However, the accurate circuits of the cerebellar cortex lobe to cerebellar DCN that play roles in sensorimotor coordination remain unclear.

The fastigial nucleus (FN), lateral (Lat), and interposed (IntP) nucleus constitutes the DCN, which are the critical output of the cerebellar system.^11^ As the oldest cerebellar nucleus throughout evolution, the FN may play a critical functional role in subcortical motor coordination.^12^ Three major neuronal classes have been identified in FN, including glutamatergic, GABAergic, and glycinergic neurons, although the proportion of each is unclear.^13^ FN sends extensive projections to multiple motor structures in the cerebral cortex to regulate motor coordination.^14^ PCs of the cerebellar vermis in both anterior and posterior lobes send GABAergic axons to innervate the FN.^15^ Therefore, elucidating the interaction between PCs and the FN may be required to successfully associate motor coordination with appropriate anatomical and functional connections. Remarkably little is known, however, about the specific cellular, molecular and network mechanisms of the PCs and FN interaction that are responsible for motor coordination.

The purpose of this study was to assess the role of the cerebellar lobe→FN circuit in subjects with ataxia. We identified a novel PC circuit in the IV/V lobe projecting to FN CaMKIIα^+^ neurons, which regulated subcortical motor coordination. It was noteworthy that biorientation defective 1 (*BOD1*) was shown to be important in the circuit, while *BOD1* deficiency decreased the number of PC GABAergic projections to FN CaMKIIα^+^ neurons, resulting in FN CaMKIIα^+^ neuronal hyperactivation and ataxia behaviors in mice. Our findings enhance our understanding of the mechanism by which the cerebellar IV/V lobe^PCs^ → FN^CaMKIIα+^ circuit regulates motor coordination.

## Results

### GABAergic input from cerebellar IV/V lobe PCs to the FN

**Fig. 1:**
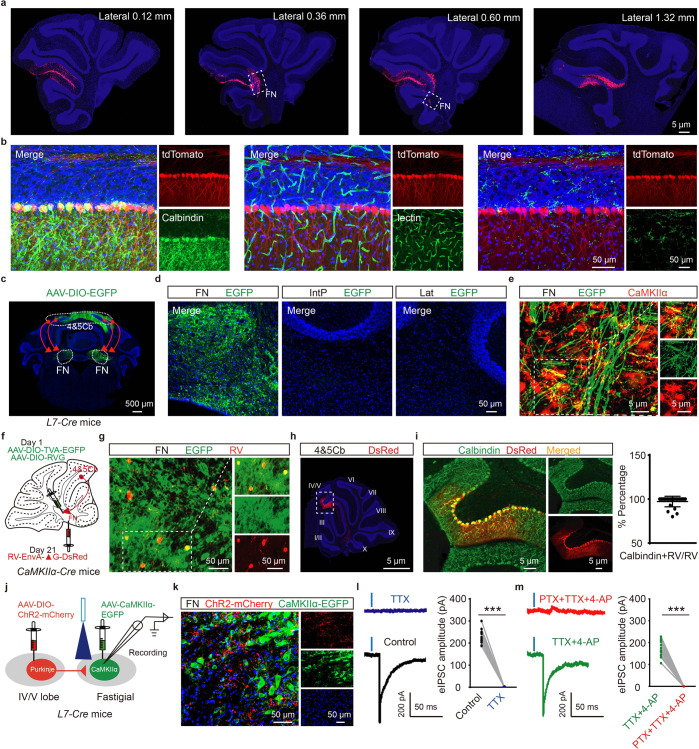
Identification of anatomical projections from cerebellar IV/V lobe to the FN. **a** Schematic injection of Red Retrobeads in FN of wild-type male mice (*n* = 3 mice per group). A section of the mouse brain cut in a sagittal plane 0.12 mm, 0.36 mm, 0.60 mm, and 1.32 mm lateral to the midline. Red Retrobeads (red), DAPI (blue). **b** Z-stack confocal images of Rosa26-tdTomato expression (Cre expression) in cerebellar IV/V lobe of *L7-Cre; Ai14* reporter mice, when co-stained with Calbindin (marker for PCs); lectin (marker for endothelial cells) and GFAP (marker for astrocytes). **c** Injection of AAV-DIO-EGFP virus in IV/V lobe of *L7-Cre* mice (*n* = 3 mice per group). **d** Anterograde tracing from IV/V lobe to the FN, IntP and Lat. 4&5Cb: cerebellar IV/V lobe, FN: fastigial nuclei, IntP: interposed nuclei, Lat: lateral nuclei. **e** Representative images showing IV/V lobe→FN PCs projections labeled with EGFP (green) and CaMKIIα-positive neurons (red) in FN. **f** FN was injected with AAV-DIO-TVA-EGFP and AAV-DIO-RVG in *CaMKIIα-Cre* mice (*n* = 3 mice per group), and 3 weeks later RV-EnvA-ΔG-DsRed was subsequently injected into the FN of the cerebellum. **g** The EGFP/DsRed double-positive cells were observed in the FN. **h** Representative images of retrograde traced (Red^+^) neurons in IV/V lobe. **i** Left: Representative images of Calbindin^+^ and Red^+^ neurons in the IV/V lobe. Calbindin marker for PCs; Right: Percentage of DsRed-labeled neurons that expressed Calbindin in IV/V lobe. **j** Schematic of IV/V lobe injection of AAV-DIO-ChR2-mCherry and FN injection of AAV-CaMKIIα-EGFP in *L7-Cre* mice and the whole-cell recordings in EGFP^+^ neurons in acute slices. **k** FN showing IV/V lobe→FN PCs projections labeled with mCherry (Red) and CaMKIIα^+^ neurons (EGFP, green). **l**, **m** Representative traces and quantification of the amplitudes of blue light-evoked IPSC traces in EGFP^+^ neurons before and after TTX, TTX + 4-AP, or TTX + 4-AP + PTX treatment (*n* = 14 cells from three mice per group; ^***^P < 0.001; unpaired two-tailed Student’s *t* test). Peak IPSC amplitudes were normalized to the baseline IPSCs recorded under control conditions. The data are presented as means ± s.e.m

Compared with the Lat and IntP nucleus, the FN, as a projection bridging hub plays a key role in motor coordination. To identify the anatomical projections from the cerebellar lobe to the FN, we injected retrograde red beads into the FN of wild-type male mice. Seven days later, robust red epifluorescence was observed in the injection position of FN and projection areas of the IV/V lobe (Fig. 1a). To confirm that PCs in lobules IV/V projected to the FN, the mice with specific Cre recombinase expression in PCs (*L7-Cre*) were used. First, we generated *L7-Cre*; *Ai14* mice to induce sufficient and specific Cre expression in PCs by crossing *L7-Cre* mice with *Ai14* reporter mice (Rosa-CAG-LSL-tdTomato-WPRE::deltaNeo). We observed that robust tdTomato expression was restricted to PCs, not in endothelial cells or astrocytes (Fig. 1b). After confirming Cre expression in PCs, we injected an anterograde adeno-associated virus encoding green fluorescent protein (AAV-DIO-EGFP) into the IV/V lobe of *L7-Cre* male mice. We observed intense EGFP labeling of synaptic terminals in the FN but not in IntP or Lat nuclei (Fig. 1c, d). Moreover, FN CaMKIIα^+^ neurons were the primary target of EGFP-labeled terminals (Fig. 1e). We further examined the PCs formed monosynaptic projections onto the FN CaMKIIα^+^ neurons by using Cre-dependent retrograde trans-monosynaptic tracing viruses. Cre-dependent helper viruses (AAV-DIO-TVA-EGFP and AAV-DIO-RVG, 1:2) were injected into the FN of *CaMKIIα-Cre* male mice. Three weeks later, we injected EnvA-pseudotyped and the rabies virus glycoprotein (RVG)-deleted rabies virus RV-EnvA-DsRed at the same coordinates (Fig. 1f). The EGFP/DsRed double-positive cells were observed in the FN, which represented the CaMKIIα^+^-neurons as starter cells in *CaMKII*α*-Cre* mice (Fig. 1g). More importantly, red fluorescent RV-infected cells appeared in the cerebellar IV/V lobe (Fig. 1h), a large population of which were calbindin-positive PCs (90.03% ± 5.116) (Fig. 1i). Thus, these data suggest that the IV/V lobe send strong Purkinje afferents to CaMKIIα^+^ neurons in the FN.

To characterize the synaptic connection from lobules IV/V to the FN, we injected AAV-CaMKIIα-EGFP into the FN and AAV-DIO-ChR2-mCherry into the IV/V lobe of *L7-Cre* mice (Fig. 1j). Histological data confirmed that ChR2-mCherry expressed in the FN, which further proved the circuit of lobules IV/V to the FN (Fig. 1k). Optogenetic activation of ChR2-expressing terminals in the FN resulted in evoked inhibitory postsynaptic currents (eIPSCs) in CaMKIIα^+^ neurons as determined by voltage-clamp recording (Fig. 1l). The eIPSCs were determined to be GABA-mediated and monosynaptic because they were completely blocked by the GABA_A_R antagonist picrotoxin (PTX) (Fig. 1m). Therefore, these results indicate that a direct monosynaptic GABAergic connection exists between the cerebellar IV/V lobe and FN CaMKIIα^+^ neurons.

### PC inhibition induces ataxia by exciting FN CaMKIIα^+^ neurons

**Fig. 2:**
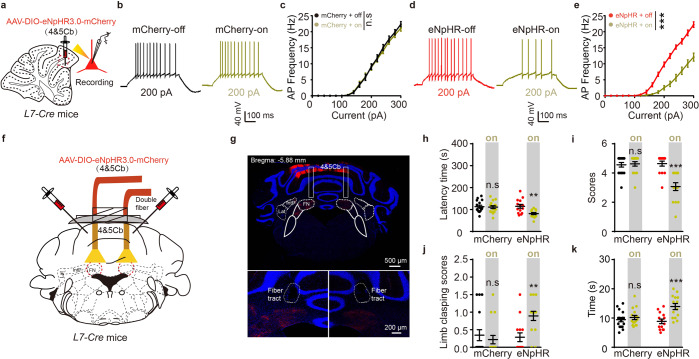
Optogenetic inhibition the circuit from IV/V lobe to FN induces ataxia-like behavior. **a** *L7-Cre* mice were injected with AAV-DIO-eNpHR3.0-mCherry or AAV-DIO-mCherry in IV/V lobe, and whole-cell recordings were performed in mCherry^+^ neurons. **b**–**e** Representative (**b**, **d**) and Quantification (**c**, **e**) of action potential (AP) traces in mCherry^+^ neurons in brain slices of IV/V lobe under 589-nm yellow light on or off (*n* = 17 cells from mCherry-injected mice; *n* = 14 cells from eNpHR-injected mice; ***P < 0.001; two-way ANOVA followed by Turkey’s multiple comparisons test). **f** Schematic of AAV-DIO-eNpHR3.0-mCherry or AAV-DIO-mCherry injection into the IV/V lobe and optical cannula implantation in the bilateral FN. **g** Representative injection site in IV/V lobe and optical fiber placement for optogenetic experiments. **h**–**k** The ataxia-related behaviors were performed (*n* = 16, 14 mice per group; **P < 0.01; ***P < 0.001; one-way ANOVA followed by Turkey’s multiple comparisons test). Quantification of the time spent on the accelerating rotarod (**h**), Quantification of the scores during runs on an elevated horizontal beam (**i**). Quantification of the limb clasping scores when suspended by the tail (**j**), Quantification of time spent on the pole (**k**). Error bars represent means ± s.e.m; ns not significant

To ascertain the functional link between the cerebellar IV/V lobe→FN circuit and motor-related behaviors, we took advantage of a new combinatorial strategy that permitted the optogenetic modulation of these projections through injection of AAV-DIO-eNpHR3.0-mCherry or AAV-DIO-mCherry to IV/V lobe. eNpHR3.0 is a fast photoactivatable electrogenic chloride (Cl^−^) pump to induce neuronal hyperpolarization upon 589-nm yellow-light laser constant stimulation. Ex vivo, brain slices containing the IV/V lobe were subjected to yellow light (constant, 30 s, 10 mW), and PCs from the IV/V lobe were subjected to whole-cell electrophysiology recording (Fig. 2a). Optogenetic stimulation of IV/V lobe PC slices decreased the firing rates in the transduced eNpHR group compared with the mCherry control group (Fig. 2b–e). To regulate eNpHR-expressing terminals in the FN, yellow light was delivered to the FN via optical fibers (Fig. 2f), and histological data confirmed mCherry expressed only in the cerebellar IV/V lobe (Fig. 2g). Inhibiting the PC terminals through yellow light in the FN reliably induced ataxia, supported by a decreased latency time on the accelerating rotarod test, lower scores on the narrow elevated beam test, higher scores on the limb-clasping test and more time spent on the pole during the pole test (Fig. 2h–k). On the other hand, inhibiting PC terminals in the IntP or Lat nucleus had no significant effect on motor coordination (Supplementary Fig. 1a–l). Altogether, these data reveal that abnormal PC neuronal input from the IV/V lobe to the FN promotes ataxia-like behavior.

To further investigate the necessity of IV/V lobe PC dysfunction for ataxia behaviors, we bilaterally injected AAV-DIO-hM4Di-mCherry (an inhibitory receptor specifically activated by the drug DREADD)^16^ into the IV/V lobe of *L7-Cre* mice. The vast majority of hM4Di-mCherry-expressing cells were located in the IV/V lobe (Supplementary Fig. 2a). The ataxia behaviors were screened three weeks after virus injection (AAV-DIO-hM4Di-mCherry or AAV-DIO-mCherry) in *L7-Cre* mice followed by clozapine-*N*-oxide administration (CNO, 1 mg/kg, i.p.). In behavioral tests, CNO impaired motor coordination and induced ataxia-like behavior (Supplementary Fig. 2b–g). Electrophysiological analysis of IV/V lobe brain slices confirmed spiking inhibition in hM4Di-expressing PCs at this time point in lobules IV/V of *L7-Cre* mice (Supplementary Fig. 2h, i). In summary, IV/V lobe PC neuronal activity is absolutely required for all aspects of ataxia behaviors.

The FN contains glutamatergic and GABAergic neuron subtypes.^17,18^ To determine the neuronal subtypes involved in ataxia behavior, the *c-fos* expression was examined.^19^ We injected AAV-DIO-eNpHR3.0 into the IV/V lobe of *L7-Cre* mice, in which optical fibers were implanted above the FN to allow the light-mediated inhibition of IV/V lobe PC inhibitory inputs in the FN. Under the rotarod test, the optogenetic inhibition of PCs increased the c-fos expression in CaMKIIα^+^ neurons (Supplementary Fig. 3a–c) but not PV^+^ neurons (Supplementary Fig. 3d). These results further indicate that the PCs in the IV/V lobe project to CaMKIIα^+^ neurons in the FN.

We next ascertain how these pathways are involved in the cerebellar IV/V lobe→FN circuit-mediated modulation of ataxia behaviors. Firing rate-dependent phase responses were recently reported to spatiotemporally organize the PC input into cerebellar nuclei.^20^ Here, electrophysiological and optogenetic approaches were used to elucidate whether alterations in the firing rate of PCs affect FN neuronal activity and mouse behavior. We injected AAV-DIO-eNpHR3.0 into the IV/V lobe of *L7-Cre* mice and implanted optical fibers into their FNs (Supplementary Fig. 3e). Consistently, single-unit recordings showed that the inhibition of PCs in lobules IV/V increased the activity of glutamatergic neurons (RS) in the FN (Supplementary Fig. 3f, g, left), whereas GABAergic neuronal (FS) activity was unchanged (Supplementary Fig. 3f, g, right). To further determine whether an increase in FN CaMKIIα^+^ neuronal activity was sufficient to drive ataxia behavior, we bilaterally injected AAV-DIO-hM3Dq-EGFP into the FN of *CaMKIIα-Cre* male mice (Supplementary Fig. 3h). Increasing the activity of FN CaMKIIα^+^ neurons by CNO (1 mg/kg, i.p.) induced ataxia as determined by behavioral analyses (Supplementary Fig. 3i–m). Therefore, the data presented herein indicate that FN hyperactivated CaMKIIα^+^ neurons are sufficient to promote ataxia behaviors.

### Deletion of *BOD1* in PCs induces ataxia behaviors and the hyperexcitability of CaMKIIα^+^ neurons in the FN

**Fig. 3:**
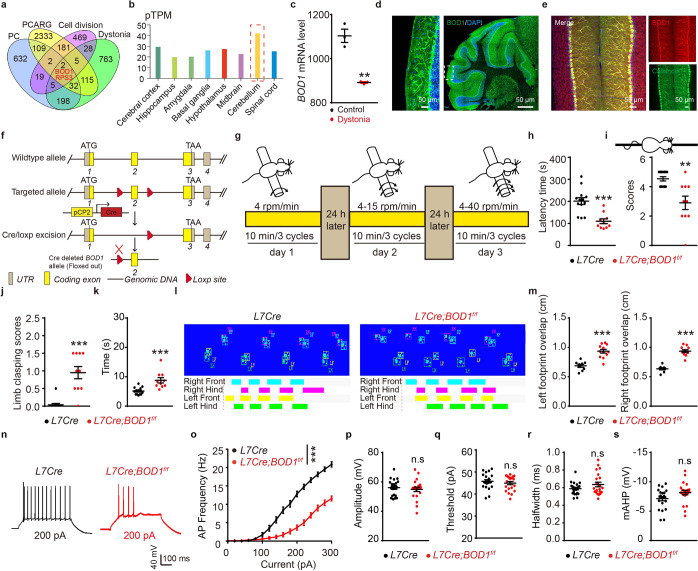
Deletion of *BOD1* in PCs induces ataxia behaviors. **a** An overlapping Venn diagram of transcriptomic differential genes in four candidate gene database. **b** mRNA expression of *BOD1* in the brain of human. **c** mRNA expression of *BOD1* in the dystonia model mice (*n* = 3 mice per group; **P < 0.01; unpaired two-tailed Student’s *t* test). **d** Representative images of BOD1 in the cerebellum. Nuclei were counterstained with DAPI (blue). **e** Representative images of BOD1 in PC layer of cerebellum of mice. Calbindin marker for PCs. **f** Schematic diagram of the strategy used to create conditional PCs-specific *BOD1* knockout (*L7-Cre; BOD1*^*f/f*^) mice. **g–k** The ataxia-related behaviors were performed in *L7-Cre* mice or *L7*-*Cre; BOD1*^*f*/*f*^ mice (*n* = 13, ten mice per group; **P < 0.01; ***P < 0.001; unpaired two-tailed Student’s *t* test). **l** Representative images of Gait footprint in *L7-Cre* mice or *L7-Cre; BOD1*^*f/f*^ mice. **m** Quantification of the left and right footprint overlap for (**l**) (*n* = 9, 12 mice per group; ***P < 0.001; unpaired two-tailed Student’s *t* test). **n** Representative AP firing of PCs evoked by current injections at 200 pA. **o** Quantification of the AP frequency of PCs by current injections from 0 to 300 pA (stepped by 20 pA) in *L7-Cre* mice or *L7-Cre; BOD1*^*f/f*^ mice for (**n**) (*n* = 22 cells from five mice per group; ***P < 0.001; two-way ANOVA followed by Turkey’s multiple comparisons test). **p**–**s** Quantification of the AP properties of PCs in *L7-Cre* mice or *L7-Cre; BOD1*^*f/f*^ mice (n = 22 cells from 5 *L7-Cre* mice, *n* = 21 cells from 5 *L7-Cre; BOD1*^*f/f*^ mice). AP amplitude (**p**), AP firing threshold (**q**), AP half-width (**r**), AP after-hyperpolarization (**s**). The error bars represent means ± s.e.m; ns not significant

To further clarify the molecular mechanism underlying ataxia mediated by abnormal cerebellar IV/V lobe PC circuit projections to FN CaMKIIα^+^ neurons, we performed a transcriptomic profile analysis of datasets to clarify candidate genes. Our research identified *BOD1* as one of two genes found in all datasets (Fig. 3a). The data from Genotype-Tissue Expression (GTEx) showed that *BOD1* is highly expressed in the cerebellum (Fig. 3b). Interestingly, the mRNA expression of *BOD1* was consistently downregulated in dystonia model mice from RNA-seq analyses data (GSE98839) (Fig. 3c).^21^ However, the role of BOD1 in the brain is largely unexplored. Immunofluorescence staining revealed that BOD1 was highly expressed in PCs and colocalized with calbindin (Fig. 3d, e). To determine whether the *BOD1* deficiency in PCs could induce ataxia, we generated conditional knockout mice in which *BOD1* was selectively ablated in cerebellar PCs (Fig. 3f and Supplementary Fig. 4a, b). In a battery of behavioral tests, the *L7-Cre; BOD1*^*f/f*^ mice consistently exhibited an ataxic phenotype (Fig. 3g–m). *BOD1* deletion, however, had no effect on the size of the cerebellum or brain weigh (Supplementary Fig. 4c–e). It also had no significant effect on the lobule morphology, PC density (Supplementary Fig. 5a–r), or electromyography (Supplementary Fig. 6a, b). To determine the effect of *BOD1* deficiency on PCs in the IV/V lobe, the membrane properties and action potentials (APs) of the PCs were recorded. Whole-cell recordings in brain slices revealed decreased IV/V lobe PC excitability in *L7-Cre; BOD1*^*f/f*^ mice (Fig. 3n, o). Further analysis of AP properties suggested unaltered parameters such as the amplitude, AP threshold, half-width, and hyperpolarization potential (Fig. 3p–s). In addition, there were no significant differences in the intrinsic membrane properties of IV/V lobe PCs between the groups at 4 weeks (Supplementary Fig. 6c–e).

To further support the role of BOD1 in modulating the excitability of PCs in the IV/V lobe and thereby regulating the function of the cerebellar IV/V lobe^PCs^ → FN^CaMKIIα+^ circuit, we examined whether the chemogenetic inhibition of the FN could ameliorate behavioral phenotypes in *L7-Cre; BOD1*^*f/f*^ mice. A mixture of an AAV expressing Cre recombinase under the control of the CaMKIIα promoter and a Cre-dependent AAV expressing hM4Di or mCherry was injected into the FN of mice (Supplementary Fig. 6f). After CNO (1 mg/kg) administration, ataxia-like behavioral analysis confirmed spiking inhibition in hM4Di-expressing CaMKIIα^+^ neurons in the FN of *L7-Cre; BOD1*^*f/f*^ mice, accompanied by an increased latency time on the rotarod test, higher scores on the suspension test, decreased scores on the limb-clasping test and a shorter time spent on the pole in the pole test (Supplementary Fig. 6g–j). Moreover, spiking inhibition in hM4Di-expressing CaMKIIα^+^ neurons in the FN of *L7-Cre; BOD1*^*f/f*^ mice decreased overt ataxia as determined by gait analysis compared to that in the control group (Supplementary Fig. 6k, l). These data suggest that BOD1 specifically modulates the excitability of PCs in the IV/V lobe and thereby regulates the function of the cerebellar IV/V lobe^PCs^ → FN^CaMKIIα+^ circuit.

### BOD1 regulates the function of the cerebellar IV/V lobe^PCs^ → FN^CaMKIIα+^ circuit

**Fig. 4:**
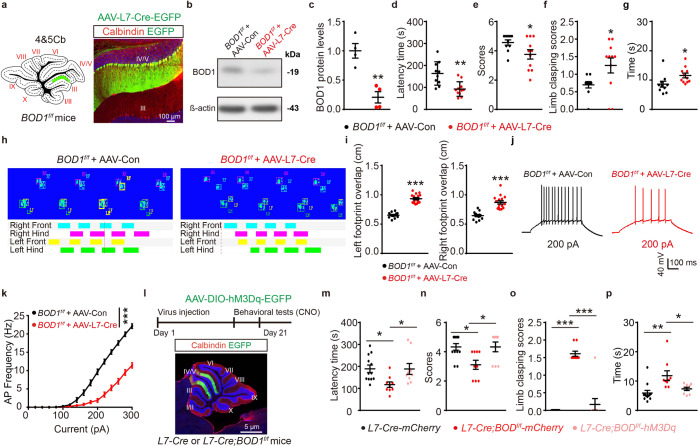
BOD1 regulates the function of cerebellar IV/V lobe^PCs^ → FN^CaMKIIα+^ circuit. **a** Schematic injection of AAV-L7-Con-EGFP or AAV-L7-Cre-EGFP and validation of EGFP in PCs of IV/V lobe. **b**, **c** Representative (**b**) and quantification (**c**) of immunoblot analysis of BOD1 protein levels in the cerebellar vermis of AAV-L7-Con-EGFP or AAV-L7-Cre-EGFP injected to IV/V lobe in *BOD1*^*f/f*^ mice (*n* = 3 mice per group; **P < 0.01; unpaired two-tailed Student’s *t* test). **d**–**g** Behavioral effects of *BOD1* deficit in IV/V lobe. After injection of AAV-L7-Con-EGFP or AAV-L7-Cre-EGFP to IV/V lobe in *BOD1*^*f/f*^ mice, the ataxia-related behaviors were performed (*n* = 10, 11 mice per group; *P < 0.05; **P < 0.01; unpaired two-tailed Student’s *t* test). **h**, **i** Representative images of Gait footprint (**h**) and quantification of the left and right footprint overlap for Gait footprint (**i**) (*n* = 13, 16 mice per group; ***P < 0.001; unpaired two-tailed Student’s *t* test). **j** Representative AP firing of PCs evoked by current injections at 200 pA. **k** Quantification of the AP frequency by current injections from 0 to 300 pA (stepped by 20 pA) in AAV-L7-Con-EGFP-injected or AAV-L7-Cre-EGFP-injected to IV/V lobe in *BOD1*^*f/f*^ mice (*n* = 24 cells from three mice per group; ***P < 0.001; two-way ANOVA followed by Turkey’s multiple comparisons test). **l** Injection of AAV-DIO-EGFP or AAV-DIO-hM3Dq-EGFP in IV/V lobe of *L7-Cre* and *L7-Cre; BOD1*^*f/f*^ mice and representative images of EGFP in PCs layer. **m**–**p** After intraperitoneal injection of CNO (1 mg/kg) to mice, the ataxia-related behaviors were performed in AAV-DIO-EGFP or AAV-DIO-hM3Dq-EGFP-injected *L7-Cre* and *L7-Cre; BOD1*^*f/f*^ mice (*n* = 12, 9, 9 mice per group; *P < 0.05; **P < 0.01; ***P < 0.001; one-way ANOVA followed by Turkey’s multiple comparisons test). The data are presented as means ± s.e.m

To determine whether the selective inhibition of *BOD1* expression in PCs of lobules IV/V is sufficient to induce ataxia, we bilaterally infused AAV-L7-Cre-EGFP into lobules IV/V of *BOD1*^*f/f*^ mice at 4 weeks of age, and histological data confirmed EGFP only expressed in lobules IV/V (Fig. 4a). The infusion markedly decreased the BOD1 expression in lobules IV/V as determined by western blot (Fig. 4b, c) and induced motor-related behavioral deficits (Fig. 4d–i) that were similar to those induced by the deletion of *BOD1* in all PCs (Fig. 3g–m). Furthermore, whole-cell recordings revealed a decreased AP frequency in the IV/V lobe PCs of AAV-L7-Cre-injected *BOD1*^*f/f*^ mice compared to AAV-Con-injected *BOD1*^*f/f*^ mice (Fig. 4j, k). These results indicate that the conditional ablation of *BOD1* in the IV/V lobe decreases the excitability of PCs, which leads to ataxia-like behaviors.

We thus hypothesized that BOD1 dysfunction might contribute to ataxia-like behaviors by regulating the activity of IV/V PCs. As *BOD1-*deficient mice displayed decreased PC excitability, we assessed whether increasing the IV/V lobe PC activity could ameliorate the deficits induced by the disruption of BOD1 function. We injected AAV-DIO-hM3Dq-mCherry or AAV-DIO-mCherry into the IV/V lobe of 4-week-old *L7-Cre* and *L7-Cre; BOD1*^*f/f*^ mice (Fig. 4l). The administration of CNO dramatically increased the latency time on the rotarod test and the suspension test scores for animals injected with AAV-DIO-hM3Dq-mCherry, whereas the AAV-DIO-mCherry injection had no effect on ataxia behaviors (Fig. 4m, n). The activation of PCs in the IV/V lobe in *L7-Cre; BOD1*^*f/f*^ mice also resulted in decreased limb-clasping scores and time on pole test (Fig. 4o, p). Taken together, these data indicate that an increase in IV/V lobe PC activity is sufficient to ameliorate ataxia-like behaviors induced by *BOD1* deficiency and that IV/V lobe PCs act downstream of cerebellar BOD1 in the regulation of these ataxia-like behaviors.

### *BOD1* deficiency decreases the PC excitability and spine density in lobules IV/V

Neuronal firing contributes to the intrinsic electrical properties in neurons as well as to excitatory and inhibitory synaptic inputs.^22^ To explore the potential mechanism by which *BOD1* deficiency affects PC activity, we recorded the miniature excitatory and inhibitory postsynaptic currents (mEPSCs and mIPSCs) in IV/V lobe PCs of 4-week-old mice. No significant changes were observed in the input impedance, membrane time constant (Tau), or capacitance in *BOD1* deficit mice (Supplementary Fig. 6c–e). However, inhibition of BOD1 expression decreased the frequency of mEPSCs but not their amplitude (Supplementary Fig. 7a–c). Consistent with the observed decreased frequency of mEPSCs, the effects of BOD1 on PC activity were blocked by the AMPA receptor inhibitor CNQX (Supplementary Fig. 7d, e) but not PTX (Supplementary Fig. 7f, g). The lack of change in the paired-pulse ratio (PPR) of evoked EPSCs and PTX-sensitive mIPSCs in PCs of *L7-Cre; BOD1*^*f/f*^ mice suggested that the decreased mEPSCs frequency was not due to a decrease in the presynaptic release probability (Supplementary Fig. 7h–l).

To determine whether the dendritic morphology and spine density contribute to decreased synaptic transmission, we injected SFV-EGFP into lobules IV/V of mice (Supplementary Fig. 7m). A significant decrease was observed in the dendritic intersection of PCs in *BOD1*-deficiency mice (Supplementary Fig. 7n, o). Imaging of PCs stained with Lucifer yellow further showed a decrease in the number of mushroom-shaped, but not stubby- or thin-shaped, spines between groups (Supplementary Fig. 7p–t), suggesting that *BOD1* deletion selectively decreased the number of mature dendritic spines in PCs.

### Transcriptional regulation cues of spine and dendrite morphogenesis by BOD1

**Fig. 5:**
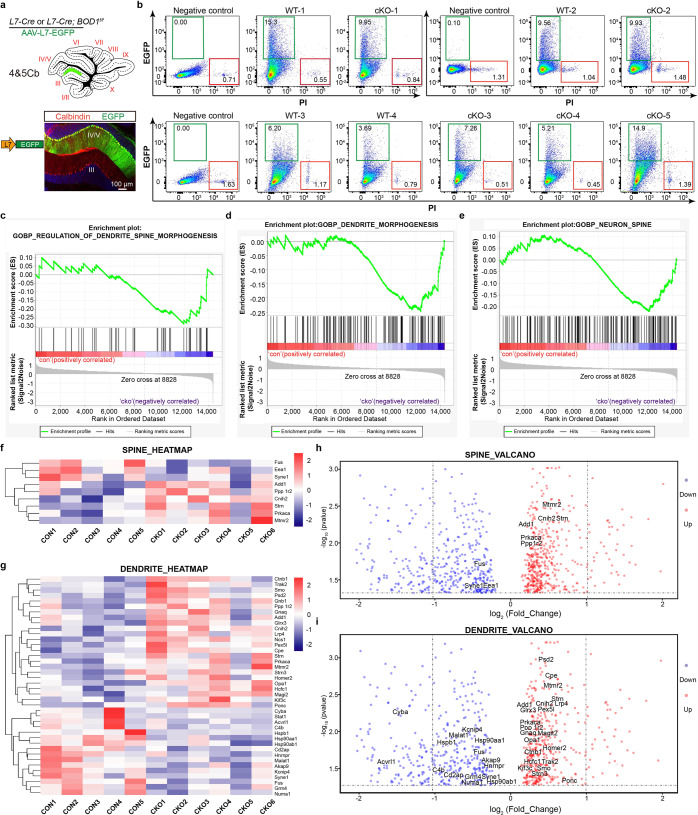
Transcriptional regulation cues of spine and dendrite morphogenesis by BOD1. **a** Schematic injection of AAV-L7-EGFP and validation of EGFP in cerebellar IV/V lobe of *L7-Cre* and *L7-Cre; BOD1*^*f/f*^ mice. EGFP enhanced green fluorescent protein, PI propidium iodide. **b** Representative flow-cytometric plot of cerebellar PCs in *L7-Cre* and *L7-Cre; BOD1*^*f/f*^ mice. **c**–**e** Enrichment plot for GO (Gene Ontology) regulation of the dendrite-spine morphogenesis response pathway (**c**), dendrite morphogenesis response pathway (**d**) and neuron-spine morphogenesis pathway (**e**). **f**, **g** Hierarchical clustering (left) and representative genes (right) regulated spine (**f**) and dendrite (**g**) between cerebellar IV/V lobe PCs in *L7-Cre* and *L7-Cre; BOD1*^*f/f*^ mice. **h**, **i** Valcano plots depicting fold change (FC) vs. P values for select spine (**h**) and dendrite (**i**) gene expression in *L7-Cre* and *L7-Cre; BOD1*^*f/f*^ mice

We next uncover the molecular mechanism that links *BOD1* deficiency to the loss of spine and dendrite. We expressed EGFP under a L7 promoter in PCs by injecting AAV-L7-EGFP into the IV/V lobe of *L7-Cre* and *L7-Cre; BOD1*^*f/f*^ mice (Fig. 5a). We collected EGFP-expressing cells by FACS and extracted the total RNA for RNA-seq (Fig. 5b). The genic profiles identified there were a significantly differences in the transcriptiomes of PCs in *L7-Cre* and *L7-Cre; BOD1*^*f/f*^ mice (Fig. 5c–i). Using an unbiased analysis of gene set enrichment for PCs sorted from cerebellar IV/V lobe in *L7-Cre* and *L7-Cre; BOD1*^*f/f*^ mice, we identified the gene sets related to spine and dendrite morphogenesis were highly correlated with *BOD1* deletion group (Fig. 5c–e). The downregulated spines genes (*Fus*, *Syne1* and *Eea1*; Fig. 5f, h) and dendrite genes (*Malat1*, *Fus*, *Hnrnpr, Syne1*; Fig. 5g, i) which play important roles in regulating spine density and dendritic branches. There were also upregulated spines genes (*Strn*, *Cnih2*) and dendrite genes (*Strn*) which negatively regulated spine and dendrite morphogenesis. To summarize, BOD1 is among the key drivers of spine and dendrite morphogenesis through regulating transcriptional profiles.

### BOD1 enrichment in IV/V lobe PCs alleviates ataxia-like behaviors

**Fig. 6:**
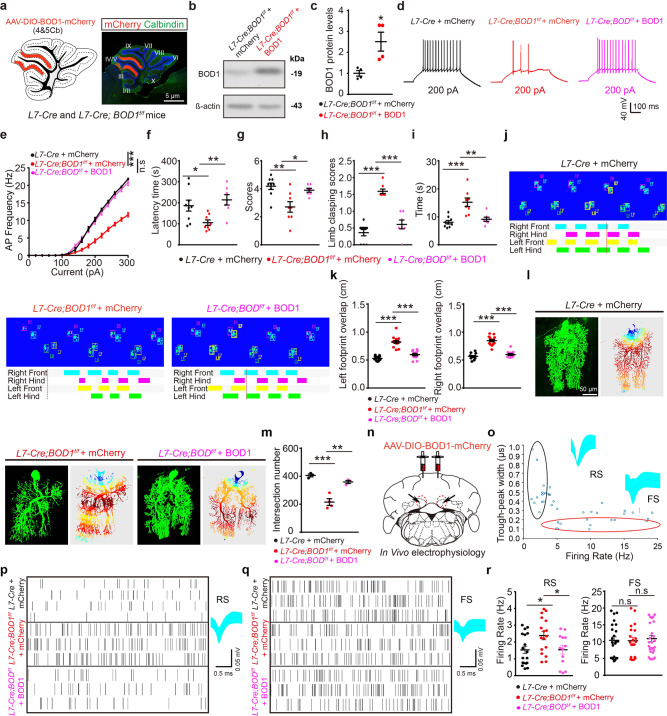
Overexpression BOD1 in the IV/V lobe of cerebellum alleviates ataxia-like behaviors in mice. **a** Representative images of mCherry (red) and Calbindin (green) double-positive neurons in cerebellar IV/V lobe of *L7-Cre* and *L7-Cre; BOD1*^*f/f*^ mice. **b**, **c** Representative image (**b**) and quantification (**c**) of BOD1 expression by western blot analysis after AAV-DIO-BOD1-mCherry or control injection (*n* = 4 mice per group; *P < 0.05; unpaired two-tailed Student’s *t* test). **d** Representative traces of AP responses to positive current injection treatments at 200 pA in mCherry^+^ neuron of *L7-Cre* and *L7-Cre; BOD1*^*f/f*^ mice with AAV-DIO-mCherry or AAV-DIO-BOD1-mCherry injection. **e** Quantification of AP frequency across 0–300 pA current injections in 20-pA steps (*n* = 25 cells from three mice per group; ***P < 0.001; two-way ANOVA followed by Turkey’s multiple comparisons test). **f**–**i** After AAV-DIO-mCherry or AAV-DIO-BOD1-mCherry injection to *L7-Cre* and *L7-Cre; BOD1*^*f/f*^ mice, ataxia-related behaviors were examined (*n* = 9, 8, 7 mice per group; *P < 0.05; **P < 0.01; ***P < 0.001; one-way ANOVA followed by Turkey’s multiple comparisons test). **j**, **k** Representative images of Gait footprint (**j**), Quantification of the left and right footprint overlap for Gait footprint (**k**). *n* = 13, 15, 12 mice per group, respectively; ***P < 0.001; one-way ANOVA followed by Turkey’s multiple comparisons test. **l** Representative images (left) and heat map (right) of PCs after 8 h of AAV-DIO-mCherry or AAV-DIO-BOD1-mCherry injection. **m** Quantification of intersection number in dendritic branches (*n* = 3 mice per group; **P < 0.01; ***P < 0.001; one-way ANOVA followed by Turkey’s multiple comparisons test). **n** Diagram depicting the experimental approach for combining viral injection and in vivo electrophysiology recording. **o** Units were classified into clusters by peak-to-valley width and firing rate. RS glutamatergic neurons, FS GABAergic neurons. **p**, **q** Representative raster plot and waveform of RS (**p**) or FS (**q**). **r** Quantification firing rate of glutamatergic (*n* = 18, 16, 13 cells from three mice) and GABAergic neurons (*n* = 23, 20, 29 cells from three mice) (*P < 0.05; one-way ANOVA followed by Turkey’s multiple comparisons test). The error bars represent means ± s.e.m; ns not significant

The above results suggest that *BOD1* deficiency induces ataxia by inhibiting the projection of PCs to the FN to induce FN CaMKIIα^+^ neuronal hyperactivity, further suggesting that ataxia can be alleviated by increasing the BOD1 expression in the PCs of lobules IV/V. AAV-DIO-BOD1-mCherry or AAV-DIO-mCherry was injected into the IV/V lobe of 4-week-old *L7-Cre* and *L7-Cre; BOD1*^*f/f*^ mice, and BOD1 expression was confirmed by immunofluorescence and western blot analyses (Fig. 6a–c). BOD1 expression increased the excitability of PCs in lobules IV/V as determined by whole-cell recording (Fig. 6d, e) and reduced ataxia as determined by behavioral analyses (Fig. 6f–k). Consistently, overexpression of *BOD1* reversed the decrease in the dendritic intersection (Fig. 6l, m). BOD1 expression in lobules IV/V also reversed the hyperactivity of FN CaMKIIα^+^ neurons in *L7-Cre; BOD1*^*f/f*^ mice (Fig. 6n–p, r), whereas the GABAergic neuronal activity was unchanged (Fig. 6n, o, q, r). These results not only support a role of projections from lobules IV/V to the FN in subjects with cerebellar ataxia but also identify the pathway as a potential therapeutic target for the disease.

## Discussion

The neural circuitry and molecular mechanisms that contribute to ataxia-like behavior remain incomplete. Until now, there have been no effective pharmacological treatment options available. In this study, we discovered a novel circuit of GABAergic PCs in distinct IV/V lobe that projects to CaMKIIα^+^ neurons in the FN to regulate motor coordination. Furthermore, transcriptomic profiling analysis revealed various cerebellar neuronal identities, and we validated that BOD1 played an important role in maintaining mature synapse density and excitability in PCs of the cerebellar IV/V lobe. BOD1 enrichment decreased the hyperexcitability of CaMKIIα^+^ neurons in the FN and eventually ameliorated ataxia behaviors. Hence, we suggest that modulation of the cerebellar IV/V lobe^PCs^ → FN^CaMKIIα+^ circuit might provide therapeutic targets for the treatment of ataxia behaviors.

To the best of our knowledge, this is the first report of this distinct cerebellar IV/V lobe regulating ataxia behaviors via a previously unmapped IV/V lobe^PCs^ → FN^CaMKIIα+^ circuit architecture. The PCs first receive the two excitatory inputs from parallel fiber and climbing fiber and the three inhibition inputs from PCs, stellate, and basket cells; they then project axons to portions of the DCN (FN, IntP, and Lat).^23,24^ Here, we identified a novel circuit of the IV/V lobe to the FN by transporting retrograde/anterograde tracers, which regulated subcortical motor coordination, but not IntP or Lat nuclei. Furthermore, we identified the projection of PCs in the IV/V lobe to CaMKIIα^+^ neurons in the FN. Inhibition of PCs in the IV/V lobe was sufficient to increase the AP frequency of CaMKIIα^+^ neurons in the FN, which were examined by 32-channel in vivo recordings.

Distinct from cerebellar IntP and Lat nuclei, neuronal firing in FN correlates with force and time derivatives of proximal limb movements.^25^ The majority of glutamatergic fastigial neurons can be further classified into various types by the expression of SPP1, SNCA, and CALB2 in subregions of FN and connected with a specific set of PCs.^26^ We herein showed that posterior FN CaMKIIα^+^-neurons were directly innervated by IV/V lobe PCs by using a Cre-dependent retrograde trans-monosynaptic tracing strategy. Consistently, we found that hyperactivation of CaMKIIα^+^ neurons in the FN through chemogenetic methods induced motor coordination defects and gait abnormalities. The direct projections from the FN to the ventrolateral periaqueductal gray (vlPAG) mediate fear memories,^27^ whereas modulation of the FN to the central lateral thalamus is sufficient for seizure control.^28^ Viewing together, divergent neural pathways connecting with the FNs mediate distinct components of the behavioral responses. Firing rate-dependent phase responses can spatiotemporally organize the PC input to cerebellar nuclei. Notably, in vivo electrophysiology recordings confirmed that yellow-light stimulation in the FN robustly activated glutamatergic neurons. These results suggest that the inhibitory inputs from the IV/V lobe to the FN trigger ataxia-like behavior mainly by affecting the activity of CaMKIIα^+^ neurons in the FN.

Our findings increase the mechanistic understanding of the involvement of key biochemical signals in cerebellar-regulated circuits in relation to ataxia-like behavior. Cerebellar ataxias are a group of sporadic or inherited disorders that have heterogeneous clinical presentations and negative impacts on daily life activities in many cases,^29^ and genes associated with cerebellar ataxia are continuously being recognized.^30^ The ablation of *Band 4.1B* and *Whirlin* leads to cerebellar PC axon pathology and motor dysfunction,^31^ and mutation of *CACNA1G* establishes a link between calcium channelopathies and cerebellar ataxias.^32^ Here, our transcriptomic profiling data identified BOD1 as a potential key molecule involved in the pathological process of cerebellar ataxia.

Consistent with converging data, we validated that *BOD1* deficiency in mouse PCs caused ataxia-like behaviors, although the deficiency did not affect the size of the cerebellum. Here, we provided causal evidence that *BOD1* deficiency decreased the mEPSC frequency in the PCs of the IV/V lobe and mature spine density. Moreover, we identified the *BOD1* deficiency-mediated transcriptional cues related to spine and dendrite morphogenesis, including downregulated genes and upregulated genes. Indeed, transcription factors play a prominent role in all facets of spine and dendrite morphogenesis.^33,34^ Hence, our transcriptional profiles data further reveal BOD1 as one of the key drivers of spine and dendrite morphogenesis. Intriguingly, overexpression of BOD1 locally in the IV/V lobe increased the firing frequency of PCs, improved ataxia-like behaviors, and augmented dendritic maturation in *L7-Cr*e; *BOD1*^*f/f*^ mice. Together, our data indicate that BOD1 plays an important role in the function of PCs and that disruption of the IV/V lobe^PCs^ → FN^CaMKIIα+^ circuit by *BOD1* deficiency mediates ataxia-like behavior in mice.

In summary, we delineated and assigned a dedicated function of the IV/V lobe^PCs^ → FN^CaMKIIα+^ circuit and revealed it to be a critical component of the motor circuitry which dysfunction induces ataxia-like behaviors. Moreover, our data suggest that decreased PC activity contributes to dysfunction in the IV/V lobe→FN circuit and motor coordination defects after the ablation of *BOD1* in the IV/V lobe. A recent study showed that cerebellar deep brain stimulation reduced PC misfiring-generated ataxia or high-amplitude action tremors.^35,36^ In this context, the present data suggest possible therapeutic avenues for combatting ataxia behaviors in patients with neurological disorders via modulation of the cerebellar IV/V lobe^PCs^ → FN^CaMKIIα+^ circuit. A further consideration is needed on how to facilitate the clinical translation of these studies, especially the development of appropriate drug treatment strategies for diverse patients with various identified and unidentified genetic etiologies.

## Materials and methods

### Antibodies, chemicals, and vectors

**Table:** 

Reagent or resource	Sources	Identifier
Antibody		
Rabbit anti-CaMKIIα	Abcam	Cat#ab22609;RRID:AB_447192
Mouse anti-Calbindin	Sigma Aldrich	Cat#C9848;RRID:AB_476894
Rabbit anti-c-fos	SYSY	Cat#226003;RRID:AB_2231974
Mouse anti-PV	Swant	Cat#PV235;RRID:AB_10000343
Mouse anti-BOD1	Hengyu Fan (Zhejiang University)	N/A
Fluorescein lycopersion esculentum lectin	Vector Laboratories	Cat#FL1171;RRID:AB_2307440
Mouse anti-GFAP	Millipore	Cat#MAB360;RRID:AB_11212597
Rabbit anti-Lucifer yellow	Thermo Fisher Scientific	Cat#A5750;RRID:AB_2536190
Alexa Fluor 488 anti-rabbit IgG	Invitrogen	Cat#A0423;RRID:AB_2335700
Alexa Fluor 488 anti-mouse IgG	Life Technologies	Cat#A21202;RRID:AB_141607
Alexa Fluor 594 anti-rabbit IgG	Life Technologies	Cat#A21207;RRID:AB_10049744
Alexa Fluor 488 anti-goat IgG	Life Technologies	Cat#A11055;RRID:AB_142672
Bacterial and virus strains		
rAAV-Ef1α-His-EGFP-2a-TVA-WPRE-pA	BrainVTA, Wuhan, China	N/A
rAAV-Ef1α-DIO-RVG-WPRE-pA	BrainVTA, Wuhan, China	N/A
RV-ENVA-ΔG-dsRed	BrainVTA, Wuhan, China	N/A
rAAV-Ef1α-DIO-EGFP-WPREs	BrainVTA, Wuhan, China	N/A
rAAV-Ef1α-DIO-hChR2(H134R)-mCherry-WPRE-hGH-pA	BrainVTA, Wuhan, China	N/A
pAAV-CaMKIIα-MCS-EGFP-3FLAG	Obio Technology, Shanghai, China	N/A
pAAV-Ef1α-DIO-eNPHR3.0-mCherry	Obio Technology, Shanghai, China	N/A
pAAV-Ef1α-DIO-mCherry	Obio Technology, Shanghai, China	N/A
rAAV-Ef1α-DIO-hM3Dq(Gq)-EGFP-WPREs	BrainVTA, Wuhan, China	N/A
pAAV-Ef1α-DIO-hM4Di(Gi)-mCherry	Obio Technology, Shanghai, China	N/A
pAAV-Ef1α-DIO-mCherry	Obio Technology, Shanghai, China	N/A
pAAV-CaMKIIα-EGFP-2A-Cre	Obio Technology, Shanghai, China	N/A
rAAV-L7-CRE-P2A-EGFP-WPRE-hGH-pA	BrainVTA, Wuhan, China	N/A
rAAV-L7-EGFP-WPRE-hGH-pA	BrainVTA, Wuhan, China	N/A
pAAV-Ef1α-DIO-mCherry-WPRE	Obio Technology, Shanghai, China	N/A
pAAV-Ef1α-DIO-mCherry-P2A-BOD1-3×Flag-WPRE	Obio Technology, Shanghai, China	N/A
pAAV-L7-mNeonGreen-2A-3Flag	Obio Technology, Shanghai, China	N/A
Chemicals, peptides, and recombinant proteins		
Red retrobeads	Lumafluor	Cat#R170
DAPI	Thermo Fisher Scientific	Cat#D1306
Picrotoxin	Tocris	Cat#1128
CNQX	Tocris	Cat#0190
DL-AP5	Tocris	Cat#0105
Tetrodotoxin (TTX)	Tocris	Cat#1078
Clozapine-N-oxide (CNO)	Sigma-Aldrich	Cat#C0832
Lucifer yellow CH lithium salt	Invitrogen	Cat#L453
Experimental models		
Mouse:*C57BL/6J* wild-type	SHANGHAI SLAC (Shanghai, China)	N/A
Mouse:*B6.Cg-Tg(CaMKIIα-Cre)T29-1Stl/J*(*CaMKIIα-Cre*)	The Jackson Laboratory	JAX:005359
Mouse:*BOD1*^*f/f*^	This paper	N/A
Mouse:*B6.Cg-Gt(ROSA)26Sor*^*tm14(CAG-tdTomato)Hze*^*/J*(*Ai14*)	The Jackson Laboratory	JAX:007914
* L7-Cre*(*pCP2-Cre*)	Ying Shen (Zhejiang University)	N/A
Oligonucleotides		
* BOD1 floxed* mouse genotyping primers: Floxed allele: Fwd: CTGTCAGCTACAGGCTGCTG; Rev: TGACCTTCTCTCCAACTGGAGG	This paper	N/A
* L7-Cre* line mouse genotyping primers: Fed: TGCCACGACCAAGTG ACA GCA ATG; Rev: ACCAGAGACGGAAAT CCA TGG CTC	This paper	N/A
Software and algorithms		
ImageJ	NIH	https://imagej.nih.gov/ij;RRID:SCR_003070
Fiji	NIH	https://fiji.sc/;RRID: SCR_002285
Prism	GraphPad	https://www.graphpad.com/scientific-software/prism/;RRID:SCR_002798
Clampit	Molecular Devices	https://www.moleculardevices.com/systems/conventional-patch-clamp/pclamp-10-software
MATLAB	The MathWorks Inc	https://www.mathworks.com/products/matlab.html;RRID:SCR_001622
R×64-3.6.2	Bell Laboratories	https://cran.r-project.org/bin/windows/base/old/3.6.2/

### Animal experiments and ethics statement

All mouse experiments were performed in accordance with the relevant guidelines for the care and use of laboratory animals of Nanjing Medical University in China. Mice were housed under a 12-h light/dark cycle and provided access to standard food and water ab libitum. All mice were acclimated to their environment for ≥1 week before the experiments.

*C57BL/6J* wild-type mice were obtained from SHANGHAI SLAC (Shanghai, China). *B6.Cg-Tg(CaMKIIα-Cre)T29-1Stl/J* (*CaMKIIα-Cre*) were obtained from The Jackson Laboratory (stock no. 007914). *BOD1*-floxed mice were crossed with *L7-Cre* mice^37^ (gift from Prof. Y Shen, Zhejiang University) to generate *L7-Cre; BOD1*^*f/f*^ mice. L7 reporter embryos were obtained by crossing *B6.Cg-Gt(ROSA)26Sor*^*tm14(CAG-tdTomato)Hze*^*/J* (*Ai14*) mice (Rosa26-tdTomato Cre reporter line, stock no. 007914; The Jackson Laboratory) with *L7*-*Cre* mice.

### Generation of the floxed *BOD1* allele

The mouse *BOD1*-floxed targeting vector was generated by Nanjing BioMedical Research Institute of Nanjing University (NBRI). The following strategy is designed firmly based on *C57BL/6J* wild-type mouse background. First, *BOD1-201* (ENSMUST00000058060.13) is selected for the recommended strategy. *BOD1-201* gene has four exons, with the ATG start codon in exon 1 and TAA stop codon in exon 3. They make LoxP sites inserted in upstream of 5′-UTR and intron 2–3 respectively by homologous recombination (Fig. 3f). When mating with cre expression allele, the sequence between two LoxP sites can be deleted in specific tissues or cells, then *BOD1* gene will be disrupted.

The genotype of this mouse line was confirmed by PCR. The primers are as follows: *BOD1*-floxed mouse (floxed allele), forward: 5′-CTGTCAGCTACAGGCTGCTG-3′ and reverse: 5′-TGACCTTCTCTCCAACTGGAGG-3′.

### Stereotaxic injection

The mice were anesthetized and viral vectors were microinfused via glass pipettes bilaterally at 300 nl per side and 50 nl min^−1^ unless otherwise specified.^38^ After microinfusion, the glass pipette was left in the brain for another 7 min to allow sufficient time for viral diffusion. For infusions of Retrobeads, the pipette remained in the brain for 10–15 min after the infusion to minimize the spread of tracers outside of regions of interest.

For monosynaptic retrograde tracing, Red Retrobeads were obtained from Lumafluor InC (R180) and microinfused unilaterally at a 200 nl volume and 50 nl min^−1^ into the FN of 4-week-old wild-type mice to retrogradely label regions that send monosynaptic projections to the FN of the cerebellum (anteroposterior: −2.8 mm; mediolateral: ±0.90 mm; dorsoventral: −3.0 mm, from lambda). rAAV-Ef1α-His-EGFP-2a-TVA-WPRE-pA (4.53 × 10^12^ viral particles ml^−1^) and rAAV-Ef1α-DIO-RVG-WPRE-pA (BrainVTA) (5.06 × 10^12^ viral particles ml^−1^) (1:2, 200 nl of helper viruses) were injected into the FN of 4-week-old *CaMKIIα-Cre* mice, and RV-ENVA-ΔG-dsRed (2.00 × 10^8^ infectious units ml^−1^) was injected 3 weeks later.

For monosynaptic anterograde tracing, rAAV-Ef1α-DIO-EGFP-WPREs (5.95 × 10^12^ viral particles ml^−1^) (BrainVTA) was injected bilaterally into the cerebellar IV/V lobe of 4-week-old *L7-Cre* mice (anteroposterior: −6.3 mm; mediolateral: ±0.48 mm; dorsoventral: −2.0 mm, from bregma).

For optogenetic manipulation, rAAV-Ef1α-DIO-hChR2(H134R)-mCherry-WPRE-hGH-pA (5.22 × 10^12^ viral particles ml^−1^) (BrainVTA) was injected into the IV/V lobe of the cerebellum, and pAAV-CaMKIIα-MCS-EGFP-3FLAG (1.47 × 10^13^ viral particles ml^−1^) (Obio Technology) was injected into the FN of 4-week-old *L7-Cre* mice. In addition, pAAV-CaMKIIα-MCS-EGFP-3FLAG (1.28 × 10^13^ viral particles ml^−1^) or pAAV-Ef1α-DIO-mCherry (1.84 × 10^13^ viral particles ml^−1^) (Obio Technology) was bilaterally microinfused into the cerebellar IV/V lobe of 4-week-old *L7-Cre* mice.

For chemogenetic manipulation, rAAV-Ef1α-DIO-hM3Dq(Gq)-EGFP-WPREs (2.65 × 10^12^ viral particles ml^−1^) or rAAV-Ef1α-DIO-EGFP-WPREs (5.95 × 10^12^ viral particles ml^−1^) (BrainVTA) was bilaterally microinfused into the cerebellar FN of 4-week-old *CaMKIIα-Cre* mice and into the cerebellar IV/V lobe of *L7-Cre* mice. For chemogenetic inhibition, pAAV-Ef1α-DIO-hM4Di(Gi)-mCherry (5.69 × 10^12^ viral particles ml^−1^) or pAAV-Ef1α-DIO-mCherry (1.84 × 10^13^ viral particles ml^−1^) (Obio Technology) was bilaterally microinfused into the cerebellar IV/V lobe of 4-week-old *L7-Cre* mice or *BOD1*^*f/f*^ mice and *L7-Cre; BOD1*^*f/f*^ mice, which were also administered pAAV-CaMKIIα-EGFP-2A-Cre (7.89 × 10^12^ viral particles ml^−1^) (Obio Technology) via their FNs.

For deletion or overexpression of *BOD1*, rAAV-L7-EGFP-WPRE-hGH-pA (5.10 × 10^12^ viral particles ml^−1^) or rAAV-L7-CRE-P2A-EGFP-WPRE-hGH-pA (5.00 × 10^12^ viral particles ml^−1^) (BrainVTA) was bilaterally microinfused into the IV/V lobe of 4-week-old *BOD1*^*f/f*^ mice. pAAV-Ef1α-DIO-mCherry-WPRE (1.64 × 10^13^ viral particles ml^−1^) or pAAV-Ef1α-DIO-mCherry-P2A-BOD1-3×FLAG-WPRE (9.99 × 10^12^ viral particles ml^−1^) (Obio Technology) was bilaterally microinfused into the cerebellar IV/V lobe of 4-week-old *L7-Cre* mice and *L7-Cre; BOD1*^*f/f*^ mice.

For sorting PCs in the cerebellar IV/V lobe, pAAV-L7-mNeonGreen-2A-3FLAG (7.63 × 10^12^ viral particles ml^−1^) (Obio Technology) was bilaterally microinfused into the IV/V lobe of 4-week-old *L7-Cre* mice and *L7-Cre; BOD1*^*f/f*^ mice.

### Immunocytochemistry

Mice were anesthetized and perfused with 4% paraformaldehyde (PFA) in phosphate-buffered saline (PBS).^39^ For immunolabeling, the 40-μm-thick sections were incubated with the primary antibodies overnight at 4 °C and then Alexa Fluor^®^-conjugated secondary antibodies for 1 h at room temperature. The confocal images were acquired under identical conditions and analyzed using ImageJ software (NIH).

### Optogenetic manipulations

Optical fibers were constructed in-house by attaching a 5-mm piece of 200-μm optical fiber (with a 0.37 numerical aperture) to a 1.25-mm zirconia ferrule (final fiber extended 3 mm beyond the ferrule). Fibers were attached with epoxy resin to ferrules, cut and polished. Mice received optical fiber implants 3 weeks after viral infusion. Optical fibers were stabilized to the skull with screws and black dental cement to minimize light leakage.^40^ The laser power at the tip of the fiber was adjusted using a Master-9 pulse stimulator (A.M.P.I.).

A 473-nm blue laser was delivered (20 Hz, 10 ms/pulse, 5 mW) to the mice injected with rAAV-Ef1α-DIO-hChR2(H134R)-mCherry-WPRE, and whole-cell recordings were performed at EGFP^+^ neurons in acute brain slices. For mice injected with pAAV-Ef1α-DIO-eNPHR3.0-mCherry, a 589-nm yellow-light laser was delivered (constant light, 30 s, 10 mW) and whole-cell recordings were performed at mCherry^+^ neurons in acute brain slices.

For the in vivo optogenetic stimulation of mice injected with pAAV-Ef1α-DIO-eNPHR3.0-mCherry, a 589-nm yellow-light laser (constant light, 30 s, 10 mW) was delivered to the IV/V lobe of the cerebellum, followed by 32-channel electrophysiological recordings.

### In vivo electrophysiological data acquisition and analysis

Neurophysiological signals were digitized using the Neuralynx Digital Lynx system via a multiplexing digital headstage, while light was delivered through the optic fiber. Spike channels were acquired at 40 kHz and bandpass filtered at 300 Hz–3 kHz before spike sorting. For spike sorting, a single unit was discriminated using principal component analysis, and neurons were separated into putative excitatory and inhibitory cells based on the firing rate and peak-valley duration.^41^

### Brain slice preparation

Postnatal 21–25-day-old male mice were anesthetized and then decapitated. The cutting solution were prepared (in mM)^42^: 75 sucrose, 87 NaCl, 2.5 KCl, 7 MgCl_2_, 25 NaHCO_3_, 1.25 NaH_2_PO_4_, 0.5 CaCl_2_, and 25 glucose. The 250-μm-thick slices were prepared and incubated in artificial cerebrospinal fluid (ACSF) for 30 min at 34 °C and then maintained at room temperature for 1 h. All external solutions were saturated with 95% O_2_/5% CO_2_. Mice for the AAV injection experiments were used 3 weeks later.

### Whole-cell recordings

Whole-cell recordings of PCs in the IV/V lobe of the cerebellum were performed using a MultiClamp 700B amplifier and 1550 A digitizer (Molecular Devices) with a recording electrode (3.5–5.5 MΩ tip resistance).^43^ Cells were held at −70 mV, and the pipette solution containing (in mM): 130 K-gluconate, 20 KCl, 10 HEPES, 0.2 EGTA, 4 Mg-ATP, and 0.5 Na_3_-GTP.

For eIPSC (light-evoked response) recording, *L7-Cre* mice were injected with AAV-ChR2-mCherry-eYFP in the IV/V lobe and AAV-CaMKII-EGFP in the FN. Recordings were obtained in EGFP^+^ neurons by stimulating ChR2^+^ terminals (20 Hz, 10 ms/pulse, 5 mW) in the FN. The monosynaptic nature of light-evoked currents was confirmed by bath application of tetrodotoxin (TTX; Tocris Bioscience, blocks sodium current) and 4-aminopyridine (4-AP).

For current-clamp recording, APs were recorded at a 500-ms suprathreshold current of 0–300 pA in 20-pA steps. We analyzed the first spike evoked by the minimum current to determine the AP properties.

The membrane time constant (Tau) was fit by an exponential function of the membrane potential change in response to rectangular hyperpolarizing current stimulation and induced a small (~3–5 mV) voltage deflection. Input resistance (Rin) was induced by a gradient hyperpolarizing current of −60-+10 pA stepped at 10 pA and was calculated from the slope of current–voltage plots in the linear phase.

To isolate mEPSCs, 50 µM PTX (Tocris Bioscience, a γ-aminobutyric acid type A receptor blocker) and 1 µM TTX were added to ACSF. To isolate mIPSCs, a bath solution containing 20 µM CNQX (Tocris; to block α-amino-3-hydroxy-5-methyl-4-isoxazolepropionic acid receptors), 50 µM DL-AP5 (Tocris; to block N-methyl-d-aspartate receptors), and 1 µM TTX was used. The holding potential for EPSC and IPSC recordings was −70 mV. Recordings were accepted under the condition of the resistance being <20 MΩ. Data were analyzed using Clampfit 10 (Molecular Devices) and MATLAB (MathWorks).

### Behavioral tests

All mice used for behavioral analyses were aged between 8 and 12 weeks and handled for 2–3 min/day for 3 days in advance. Rest periods of at least 1 day were utilized between tests.

For all experiments in which mice injected with pAAV-Ef1α-DIO-mCherry or pAAV-Ef1α-DIO-eNPHR3.0-mCherry were subjected to optogenetic stimulation, the light stimulation began once the mice met the 80% criterion during the initial association portion of the task. The mice then performed three additional initial association trials with light stimulation before the rule shift portion of the task began. Light stimulation did not alter the performances or behaviors of the mice during these three extra trials of the initial association.

For all experiments in which mice injected with rAAV-Ef1α-DIO-hM3Dq(Gq)-EGFP or pAAV-Ef1α-DIO-hM4Di(Gi)-mCherry were subjected to chemogenetic stimulation, CNO (1 mg/kg, C0832) was intraperitoneally injected for 30 min, then the mice were used for designed behavioral analysis_._

#### Rotarod test

The rotarod tests were conducted as described previously to measure the learning of skilled movements and motor coordination.^44^ The animals were trained on 2 successive days to run on a slowly accelerating rotarod (day 1: 4 rpm/min; day 2: 4–15 rpm/min) for 10 min each. The following day, the animals were tested at 4–40 rpm/min three times each.

#### Elevated beam balancing test

The beam balancing test was used as an additional test for motor coordination.^45^ Mice were placed onto a 2-mm-long horizontal rope beam and motivated to walk along it. The following scoring system was utilized: 5.0, run along the beam quickly; 4.0, run along the beam slowly; 3.0, three limbs clinging to the rod; 2.0, two limbs clinging to the rod; 1.0, one limb clinging to the rod; and 0.0, falling off the rod.

#### Limb-clasping test

Mice were scored when suspended by the tail for 1 min.^46^ The following scoring system was utilized: 2.0, 4-limb feet-clasping posture; 1.5, 3-limb feet-clasping posture; 1.0, 2-limb feet-clasping posture; 0.5, 1-limb feet-clasping posture; and 0.0, held limbs outward.

#### Pole test

The mouse was placed on a vertical pole with the head up (pole was 55 cm high and 1 cm in diameter).^47^ Meanwhile, a ball of yarn was placed on top of the pole to prevent mice from climbing up. The incubation period during which the mouse climbed from the top to the bottom of the vertical pole was recorded. Afterward, the training experiment was repeated three times continuously. The incubation period was examined three times continuously 24 h after the training experiment. During the experiment, the rod was cleaned before experimenting with another mouse.

#### Footprint test

Using a Catwalk system (Noldus), mice were trained for two days and then allowed to run along a glass plate runway (1.5-m-long, 15-cm-wide, 30-cm-high), and a “safe house” was at the end. The ceiling of runway is installed with a red light, and the long edge of the glass plate is illuminated with a green light (green intensity threshold 0.18 and camera gain 20.74). When the paws of mice touched the glass plate, the light will be reflected internally and captured by a high-speed video camera. The dynamic and static gait parameters are provided by CatWalk software. The left and right footprint overlap are analyzed using ImageJ software.

### Western blot analysis

Cerebellar vermis tissues were dissected and homogenized in lysis buffer as previously described.^48^ Equivalent amounts of protein were loaded onto 10–12% SDS-polyacrylamide gels and transferred to PVDF membranes (Millipore) for 2 h. The blots were probed with anti-BOD1 (1:1000, gift from Hengyu Fan, Zhejiang University) and anti-β-actin (1:10,000, A5441, Sigma Aldrich) primary antibodies at 4 °C overnight and then incubated with HRP-conjugated secondary antibodies. The proteins were visualized by an enhanced chemiluminescence detection system (Amer sham Life Science). The protein band densities were quantified using ImageJ software (US National Institutes of Health) and normalized to that of actin.

### Dendritic analysis

To analyze the dendritic intersection number, recombinant Semliki Forest virus (SFV) was constructed in combination with enhanced GFP (eGFP) as described previously.^49^ For the delivery of SFV, *L7-Cre; BOD1*^*f/f*^ and *L7-Cre* mice were anesthetized with an isoflurane gas/oxygen mixture (2%) in the stereotaxic frame (RWD Life Science) and were injected with 300 nl of recombinant SFV (2.3E6 FFU/ml) into lobules IV/V of the cerebellar vermis at a rate of 50 nl/min using a glass pipette. Eighteen hours later, the injected animals were decapitated for imaging analysis.

### Morphological spine analysis

Intracellular Lucifer yellow labeling was performed as described previously.^50^ In brief, an aqueous solution of 6% (w/v) Lucifer yellow CH lithium salt (L453, Invitrogen) was injected via a micromanipulator into PCs in 250-μm-thick brain slices under the surveillance of a Nikon FN1 fluorescence microscope (Nikon, Tokyo, Japan) with ×40 water immersion objective. The sharp glass pipettes with the dye were further iontophoretically injected at a constant current of −5 nA for 120 s after pipette penetration into the PCs of the IV/V lobe (ION-100T, Dagan, CA, USA).

After dye injection, the brain slices were fixed in 4% PFA for 3 days, washed with PBS three times, then incubated with a rabbit anti-Lucifer yellow polyclonal antibody and Alexa 488-labeled anti-rabbit IgG for spine imaging.

### Fluorescence-activated cell sorting of PCs

PCs were isolated from 4-week-old *L7-Cre; BOD1*^*f/f*^ and control mice injected with AAV-L7-EGFP. Briefly, the cerebellar vermis was dissected, then digested with trypsin-EDTA (0.1%) at 37 °C for 10 min. Digestion was stopped by adding Fetal Bovine Serum (FBS). After being resuspended in Ca^2+^- and Mg^2+^-free Hank’s balanced salt solution (HBSS) and adequate centrifugation at 2000×*g* for 5 min, PCs were counted under a microscope. After filtration 1 × 10^5^ cells were collected and resuspended in fluorescence-activated cell sorting (FACS) buffer. Propidium iodide (PI) viability dye (Vazyme) was added to the suspension 5 min before sorting. FACS was performed utilizing a FACS Aria Fusion system (BD Biosciences).^51^ EGFP^+^PI^-^ cells were identified as PCs and collected for further analysis.

### High-throughput mRNA sequencing

The PCs from fluorescence-activated cell sorting was used for RNA-seq. Total RNA of PCs in cerebellar IV/V lobe from mice were extracted using TRIzol Reagent (Invitrogen) and RNA quality was evaluated using the Agilent 2100 Bioanalyzer (Agilent Technologies, USA) and NanoDrop (Thermo Fisher Scientific Inc).^52^ Qualified samples, which had an RIN value >6.5 were used for library preparation. Sequencing library preparations were constructed and performed using Poly (A) mRNA Magnetic Isolation Module. Next, the first-strand cDNA, the second-strand cDNA, and the purified double-strand cDNA were synthesized as previously described. Sequencing was carried out using a 2 × 150 bp paired-end (PE) configuration and analyzed by GENEWIZ.

### Statistical analysis

Error bars in the figures were shown as the means ± SEM. Statistical differences were determined using the unpaired two-sided Student’s *t* test for two independent groups or one-way ANOVA followed by Tukey’s post hoc test for more than two groups or two-way ANOVA (genotype × trial) for two groups with two factors.

## Supplementary information

Supplementary Material

## Data Availability

Datasets generated in this study using RNAseg have been deposited at the GEO database under accession code “GSE199327”. All data are available in the main text or supplementary materials.
