# Supplementary material for: BOD1 regulates the cerebellar IV/V lobe-fastigial nucleus circuit associated with motor coordination
Source: Signal Transduct Target Ther. 2022 Jun 1;7:170. doi: 10.1038/s41392-022-00989-x (PMC9156688; doi:10.1038/s41392-022-00989-x)
Supplement: Supplementary file 1 — Supplementary Material [file 41392_2022_989_MOESM1_ESM.docx]

**Supplementary Materials for**

**BOD1 regulates the cerebellar IV/V lobe-fastigial nucleus circuit associated with motor coordination**

Xiu-Xiu Liu^1^, Xing-Hui Chen^2,3^, Zhi-Wei Zheng^2^, Qin Jiang^2^, Chen Li^1^, Lin Yang^2,3^, Xiang Chen^1^, Xing-Feng Mao^1^, Hao-Yang Yuan^2^, Li-Li Feng^1^, Quan Jiang^3^, Wei-Xing Shi^4^, Takuya Sasaki^5^, Kohji Fukunaga^5^, Zhong Chen^3^, Feng Han^1,6,7*^, Ying-Mei Lu^2,6*^

^1^Key Laboratory of Cardiovascular & Cerebrovascular Medicine, Drug Target and Drug Discovery Center, School of Pharmacy, Nanjing Medical University, Nanjing 211166, China.

^2^Department of Physiology, School of Basic Medical Sciences, Nanjing Medical University, Nanjing 211166, China.

^3^Institute of Pharmacology and Toxicology, College of Pharmaceutical Sciences, Zhejiang University, Hangzhou 310058, China.

^4^Department of Pharmaceutical and Administrative Sciences, Loma Linda University School of Pharmacy, Loma Linda, CA, 92350, USA; Department of Basic Sciences, Loma Linda University School of Medicine, Loma Linda, CA, 92350, USA.

^5^Department of Pharmacology, Graduate School of Pharmaceutical Sciences, Tohoku University, Sendai 980-8578, Japan.

^6^Institute of Brain Science, the Affiliated Brain Hospital of Nanjing Medical University, Nanjing 210029, China.

^7^Gusu School, Nanjing Medical University, Suzhou Municipal Hospital, The Affiliated Suzhou Hospital of Nanjing Medical University, Suzhou, 215002, China.

^*^Correspondence author, E-mail addresses: Feng Han ([fenghan169@njmu.edu.cn](mailto:fenghan169@njmu.edu.cn)); Ying-Mei Lu ([lufx@njmu.edu.cn](mailto:lufx@njmu.edu.cn))

**This PDF file includes:**Supplementary Materials and Methods
Figures. S1 to S7

**Supplementary materials and methods**

**Stereotaxic injection**

For chemogenetic inhibition, pAAV-Ef1α-DIO-hM4Di(Gi)-mCherry (5.69 × 10^12^ viral particles ml^-1^) or pAAV-Ef1α-DIO-mCherry (1.84 × 10^13^ viral particles ml^-1^) (Obio Technology, Shanghai, China) was microinfused bilaterally into the cerebellar IV/V lobe of 4-week-old *L7-Cre* mice or *BOD1^f/f^* mice and *L7-Cre; BOD1^f/f^* mice (anteroposterior: -6.3 mm; mediolateral: ±0.48 mm; dorsoventral: -2.0 mm, from bregma). CNO (1 mg/kg, C0832) was intraperitoneally injected for 30 min, and the mice were then used for behavioral analysis.

**Electromyography analysis**

The electromyograms (EMGs) of leg muscles were recorded as described before^1^. The gastrocnemius muscles of mice left hind limbs was exposed via a left hind limb incision. The gastrocnemius muscle was then hung on a pair of paralleled silver electrodes. The spontaneous muscle electrical activity was recorded and amplified with an AD/CD differential amplifier (model DP-304, Warner Instruments, Hamden, CT). The signals between 100Hz and 3000Hz were filtered and saved for further analysis.

**Supplementary figures**

**
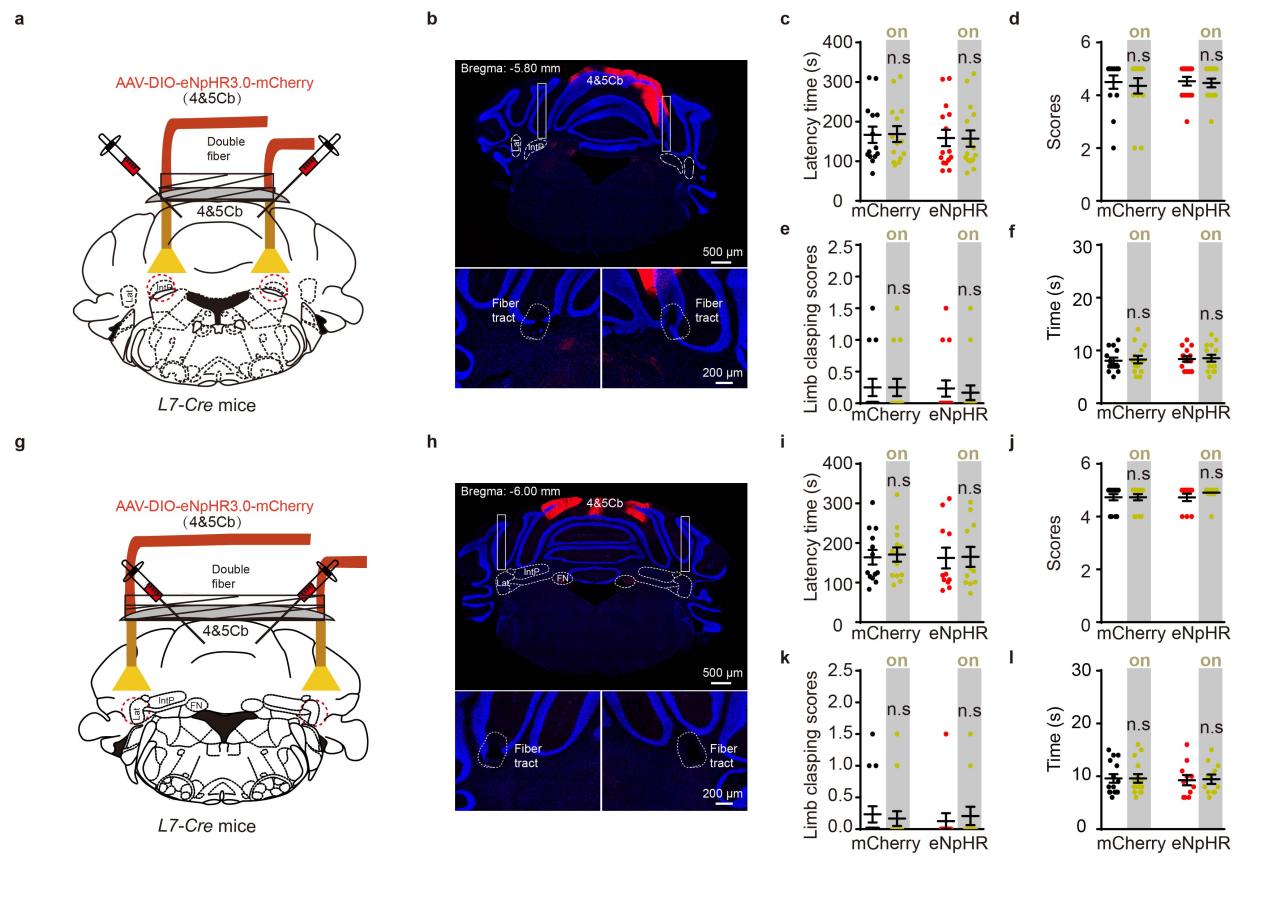
Figure S1. Behavioral effects of optogenetic inhibition the IV/V lobe to IntP or Lat circuit.**

**a** AAV-DIO-eNpHR3.0-mCherry or AAV-DIO-mCherry were injected into IV/V lobe, and optical fibers were implanted above the bilateral IntP of cerebellum in *L7-Cre* mice. **b** Represented images of mCherry expressed in the IV/V lobe of cerebellum in *L7-Cre* mice, and optical fibers were implanted above the bilateral IntP. **c-f** The ataxia-related behaviors were assessed (n=14,15 mice per group, respectively; one-way ANOVA followed by Turkey’s multiple comparisons test). **g** AAV-DIO-eNpHR3.0-mCherry or AAV-DIO-mCherry were injected into IV/V lobe, and optical fibers were implanted above the bilateral Lat of cerebellum in *L7-Cre* mice. **h** Represented images of mCherry expression in the IV/V lobe of cerebellum in *L7-Cre* mice, and optical fibers were implanted above the bilateral Lat. **i-l** The ataxia-related behaviors were assessed (n=15, 11 mice per group; one-way ANOVA followed by Turkey’s multiple comparisons test). Error bars represent means ± s.e.m; ns, not significant.

**
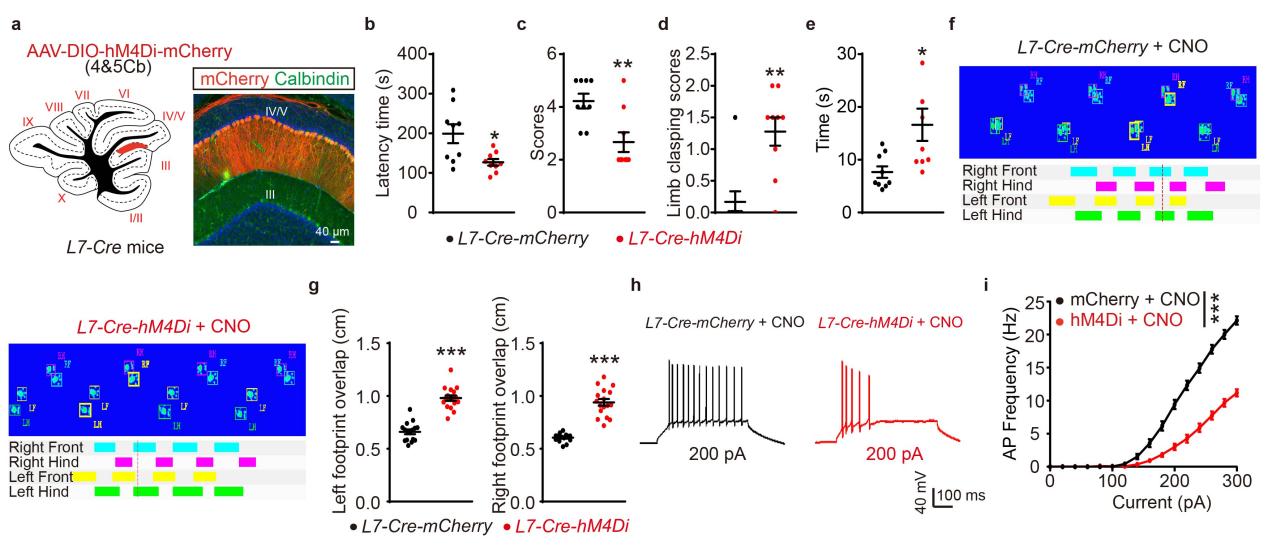
Figure S2. Chemogenetic inhibition the IV/V lobe to FN circuit promotes ataxia behavior.**

**a** Schematic injection of AAV-DIO-mCherry or AAV-DIO-hM4Di-mCherry in PCs of IV/V lobe in *L7-Cre* mice. Represented image of mCherry (red) and Calbindin (green) double-positive neurons in IV/V lobe (right). **b-e** Effect of chemogenetic inhibition on ataxia-related behaviors were assessed after AAV-DIO-mCherry or AAV-DIO-hM4Di-mCherry injection (n=9,9 mice per group; **P*<0.05; ***P*<0.01; unpaired two-tailed Student’s *t* test). **f** Representative images of Gait footprint. **g** Quantification of the left and right footprint overlap (n=16, 17 mice per group; ****P*<0.001; unpaired two-tailed Student’s *t* test). **h** Representative AP firing of PCs evoked by current injection at 200 pA. **i** Quantification of the AP frequency by current injections from 0 to 300 pA, stepped by 20 pA (n=21 cells from 3 AAV-DIO-mCherry-injected *L7-Cre* mice, n=28 cells from 3 AAV-DIO-hM4Di-mCherry-injected *L7-Cre* mice per group; ****P*<0.001; two-way ANOVA followed by Turkey’s multiple comparisons test). Error bars represent means ± s.e.m.

**
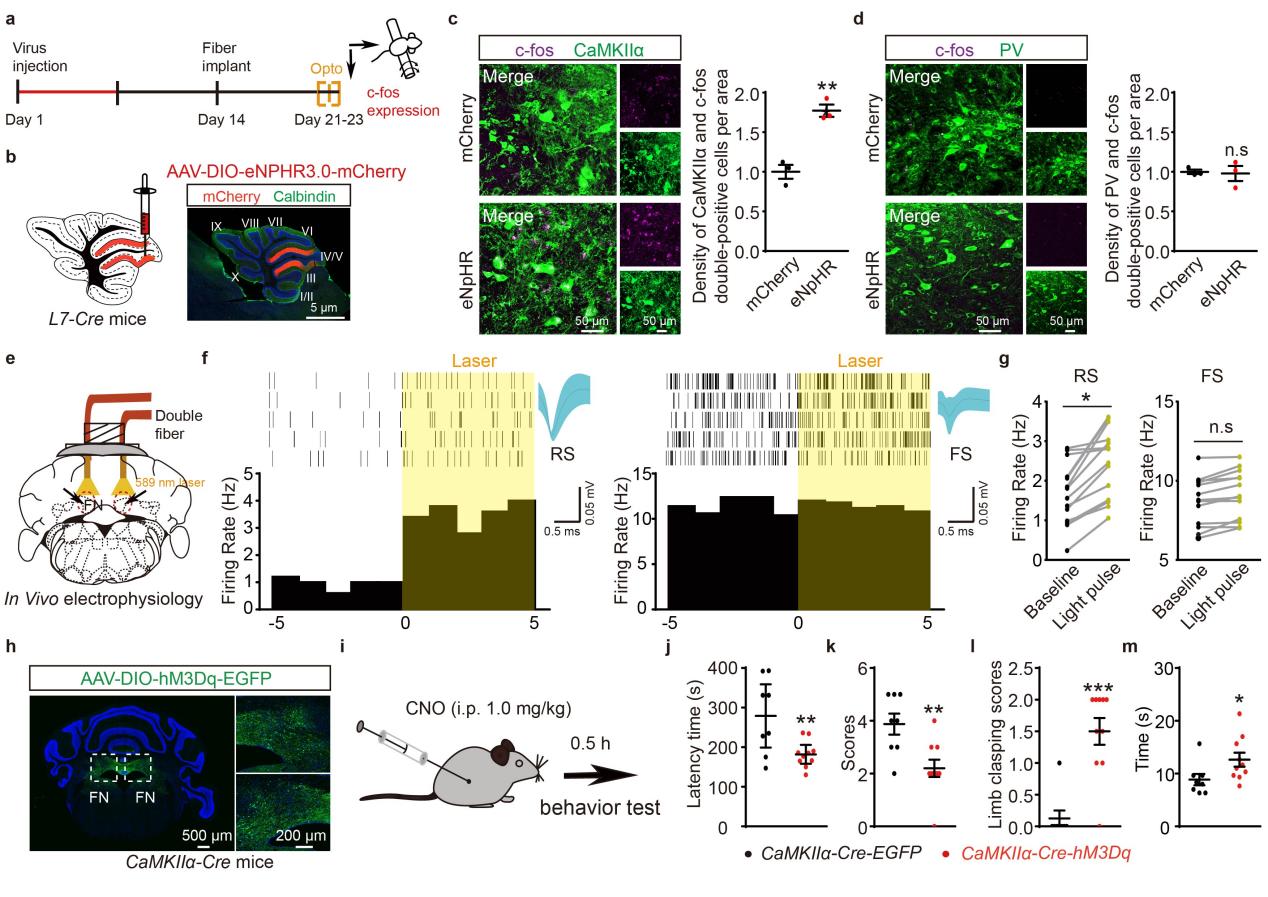
**

**Figure S3. Hyperactivated CaMKIIα^+^ neurons in FN are related to the onset of ataxia-like behavior.**

**a** Diagram of virus injection and experimental procedure in mCherry-injected or eNpHR-injected *L7-Cre* mice. **b** Schematic injection of AAV-DIO-mCherry or AAV-DIO-eNpHR3.0-mCherry in IV/V lobe in *L7-Cre* mice (left) and representative images of mCherry (red) and Calbindin (green) double-positive neurons in the IV/V lobe (right). DAPI (blue). **c, d** Representative confocal images and quantification of the c-Fos/CaMKIIα double-positive neurons (**c**) and c-Fos/PV double-positive PV neurons (**d**) in the FN after optogenetically activated eNpHR-expressing axon terminals from the IV/V lobe (n=3 mice per group; ***P* < 0.01; unpaired two-tailed Student’s *t* test). **e** Schematic diagram for optogenetic manipulation of the circuit with virus of eNpHR or mCherry injection into the IV/V lobe, optical cannula and optical fibers 32-channel recording electrodes implantation in the bilateral FN in *L7-Cre* mice. **f** IV/V lobe→FN neurons displaying photoresponses *in vivo* (bin width, 1 s). Representative raster plot, waveform of glutamatergic neurons (RS) and GABAergic neurons (FS). **g** Quantification firing rate of glutamatergic and GABAergic neurons (left: n=14 cells, right: n=13 cells; **P* < 0.05; unpaired two-tailed Student’s *t* test). **h** Schematic injection of AAV-DIO-hM3Dq-EGFP or AAV-DIO-EGFP to FN in *CaMKIIα-Cre* mice. **i-m** After intraperitoneal injection of CNO (1 mg/kg) to mice, the ataxia-related behaviors were performed in EGFP-injected or hM3Dq-injected *CaMKIIα-Cre* mice. n=8, 10 mice per group; **P* < 0.05; ***P* < 0.01; ****P* < 0.001; unpaired two-tailed Student’s *t* test. The error bars represent means ± s.e.m; ns, not significant.

**
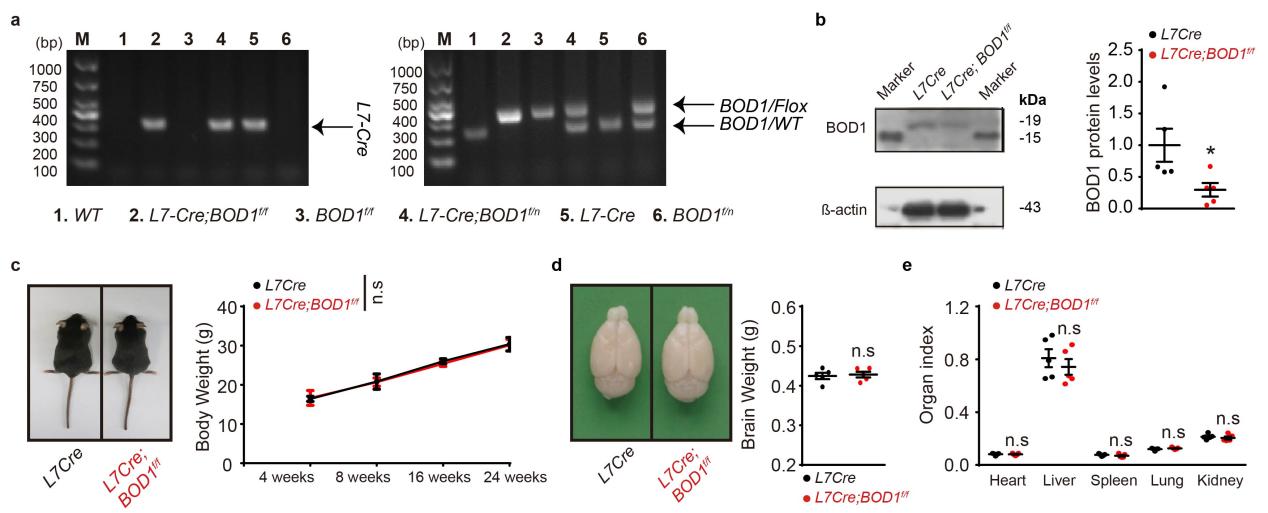
**

**Figure S4. Generation and basal physiological parameters of the *L7-Cre; BOD1^f/f^* mice.**

**a** Genotyping of *L7-Cre; BOD1^f/f^* mice. The *L7-Cre* primers generated a 400-bp product. The *BOD1* primers generated a 300-bp product in the loxP-flanked allele or a 200-bp product in the wild-type allele. **b** Representative (left) and quantification (right) of BOD1 expression by western blot analysis in cerebellar vermis of *L7-Cre* and *L7-Cre; BOD1^f/f^* mice (n=5, 5 mice per group; **P*<0.05; unpaired two-tailed Student’s *t* test). **c** Body size in 4-week-old *L7-Cre* and *L7-Cre; BOD1^f/f^* mice (left) and weight of *L7-Cre* and *L7-Cre; BOD1^f/f^* mice in different age (right) (n=3 mice at 4, 8, 16, 24 weeks per group; two-way ANOVA followed by Turkey’s multiple comparisons test). **d** Brain size (left) and weight (right) in 4-week-old *L7-Cre* and *L7-Cre; BOD1^f/f^* mice (n=5, 6 mice at 4-week-old per group; unpaired two-tailed Student’s *t* test). **e** Organ-to-body weight ratios of *L7-Cre;BOD1^f/f^* mice (n=5, 6 mice at 4-week-old per group; unpaired two-tailed Student’s *t* test). Summary graphs show means ± s.e.m; ns, not significant.

**
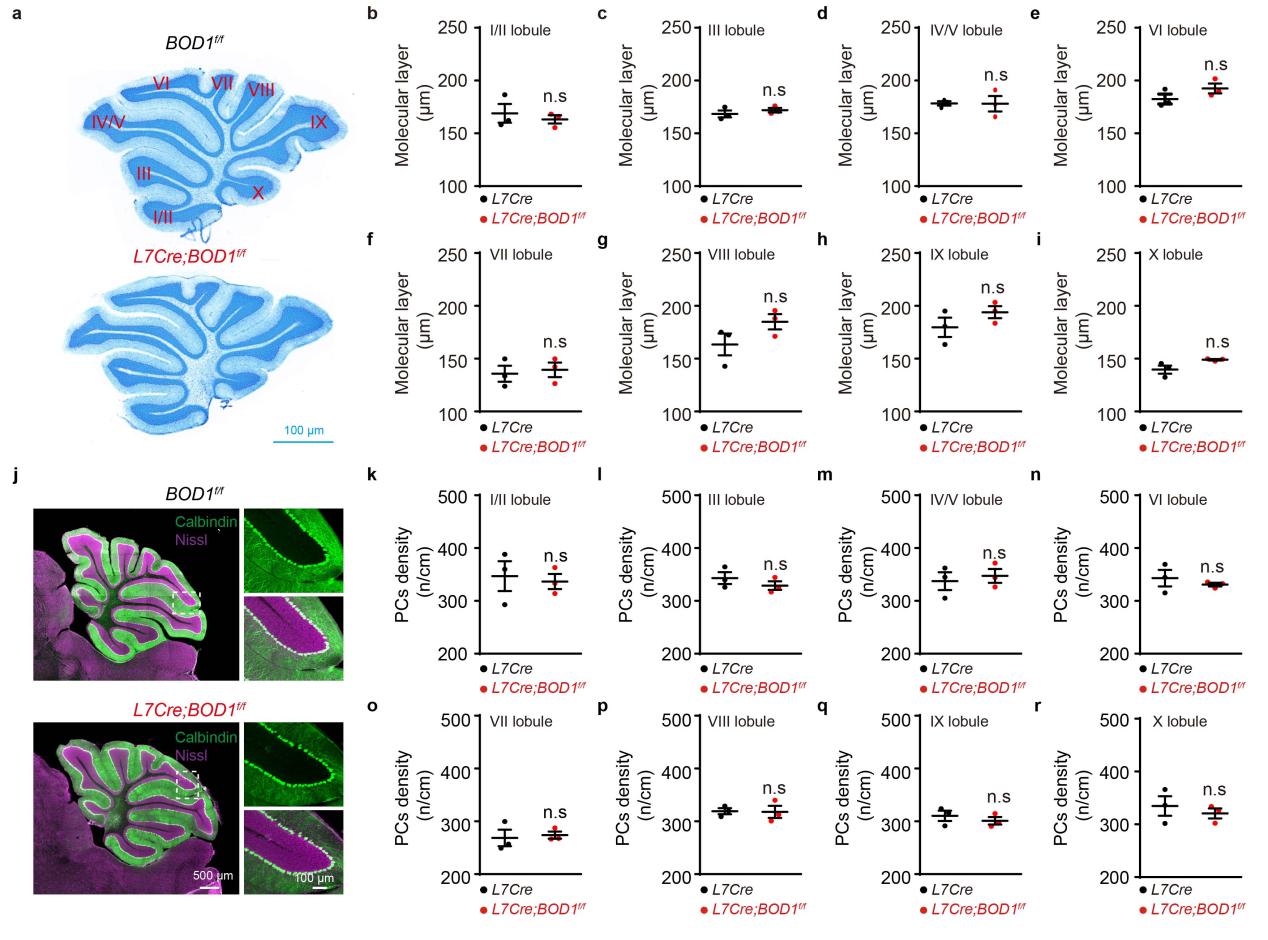
Figure S5. *BOD1* deletion in PCs has no effects on molecular layer and PCs density in cerebellum.**

**a** Representative images of cerebellum by Nissl staining in *L7-Cre* and *L7-Cre; BOD1^f/f^* mice. **b-i** Quantification of molecular layer in I-X lobe of cerebellum (n=3 mice per group; unpaired two-tailed Student’s *t* test). **j** Representative images of calbindin^+^ neurons in cerebellum. Calbindin marker for PCs. **k-r** Quantification of PCs density in I-X lobe of cerebellum (n=3 mice per group; unpaired two-tailed Student’s *t* test). Error bars represent means ± s.e.m; ns, not significant.

**
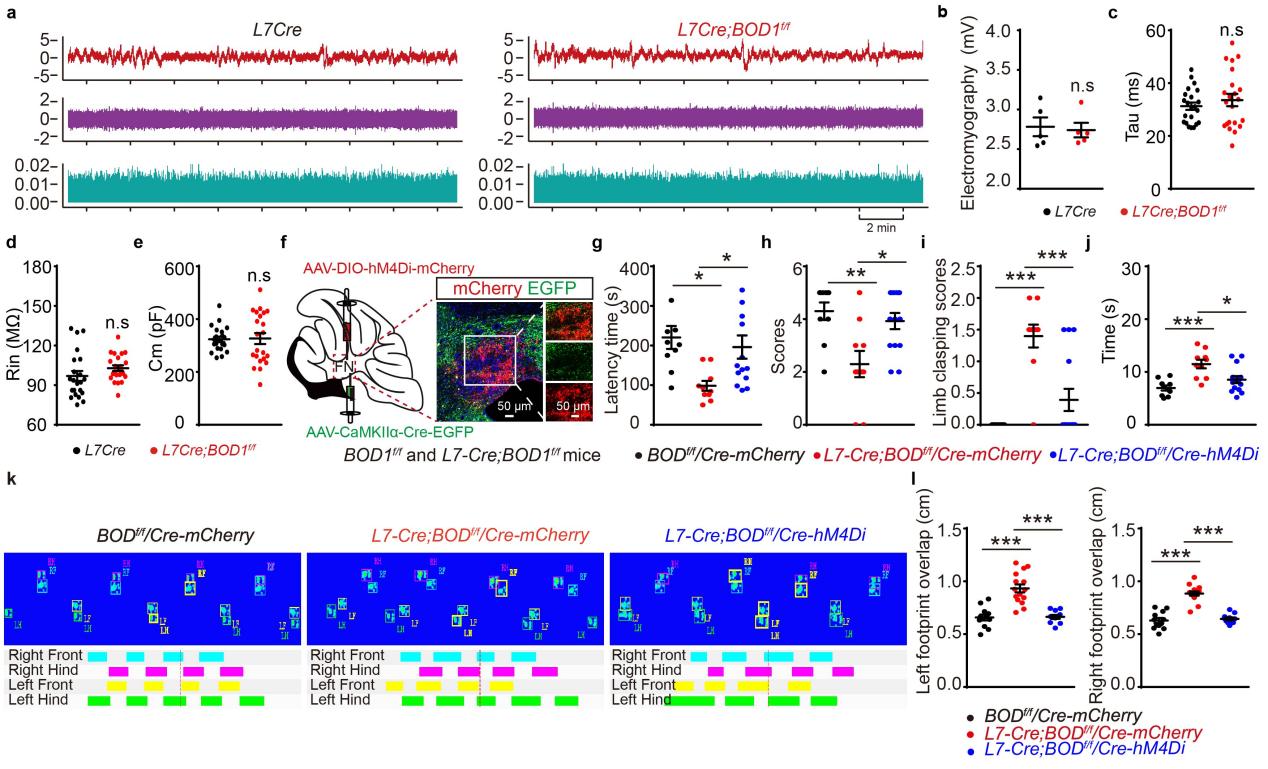
Figure S6. Inhibition the FN CaMKIIα^+^ neurons in *L7-Cre; BOD1^f/f^* mice ameliorates ataxia behavior.**

**a, b** Representative (**a**) and quantification (**b**) of electromyography recordings in *L7-Cre* and *L7-Cre; BOD1^f/f^* mice (n=5 mice per group; unpaired two-tailed Student’s *t* test). **c-e** Electrophysiological properties of PCs in cerebellar IV/V lobe by whole-cell recording. Tau (**c**), input resistance (Rin) (**d**) and membrane capacitance (Cm) (**e**) in *L7-Cre* and *L7-Cre; BOD1^f/f^* mice (n=3 mice per group; unpaired two-tailed Student’s *t* test). **f** Schematic injection of AAV-DIO-mCherry or AAV-DIO-hM4Di-mCherry mixed with AAV-CaMKIIα-EGFP and representative images of mCherry and EGFP in FN. **g-j** The ataxia-related behaviors were performed after AAV-DIO-mCherry or AAV-DIO-hM4Di-mCherry mixed with AAV-CaMKIIα-EGFP injections (n=10, 10, 14 mice per group; **P* < 0.05; ***P* < 0.01; ****P* < 0.001; one-way ANOVA followed by Turkey’s multiple comparisons test). **k, l** Representative images of Gait footprint (**k**), Quantification of the left and right footprint overlap (**l**) (n=12,15,11 mice per group; ****P*<0.001; one-way ANOVA followed by Turkey’s multiple comparisons test). The error bars represent means ± s.e.m; ns, not significant.


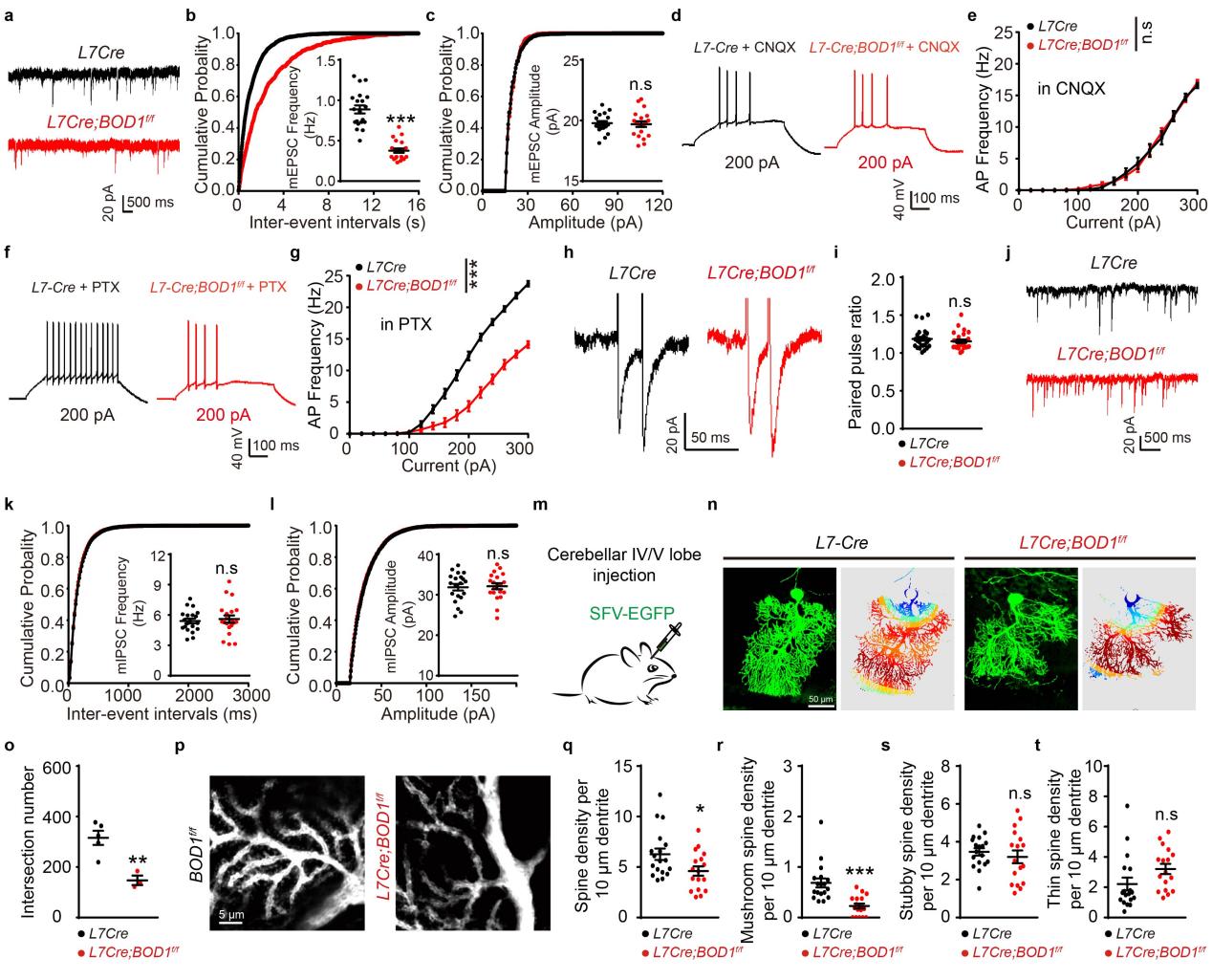


**Figure S7. *BOD1* deficit in PCs attenuates dendritic spine density in IV/V lobe.**

**a** Representative mEPSC traces recorded in a whole-cell configuration of PCs in *L7-Cre* and *L7-Cre; BOD1^f/f^* mice. **b, c** Cumulative probability plots summarizing the mean mEPSC inter-event intervals (**b**) and mEPSC amplitude (**c**). The insets depict the average mEPSC frequencies and amplitudes (n=20 cells from 3 mice per group; ****P* < 0.001; unpaired two-tailed Student’s *t* test). **d** Representative traces of AP responses to positive current injection treatment at 200 pA with CNQX (20 µM) in *L7-Cre* and *L7-Cre; BOD1^f/f^* mice. **e** Quantification of AP frequency in *L7-Cre* and *L7-Cre; BOD1^f/f^* mice with CNQX across 0-300 pA current injections in 20-pA steps (n=26 cells from 5 mice per group). **f** Representative traces of AP responses to positive current injection treatment at 200 pA with PTX (50 µM) in *L7-Cre* and *L7-Cre; BOD1^f/f^* mice. **g** Quantification of AP frequency in *L7-Cre* mice and *L7-Cre; BOD1^f/f^* mice with PTX across 0-300 pA current injections in 20-pA steps (n = 31 cells from 5 mice per group; ****P* < 0.001; two-way ANOVA followed by Turkey’s multiple comparisons test). **h** Representative traces showing paired-pulse ratio (PPR) in the IV/V lobe of *L7-Cre* and *L7-Cre; BOD1^f/f^* mice. **i** Quantification analyses of PPR (n=26 cells from 3 mice per group; unpaired two-tailed Student’s *t* test). **j** Representative mIPSC traces recorded in a whole-cell configuration of PCs in *L7-Cre* and *L7-Cre; BOD1^f/f^* mice. **k, l** Cumulative probability plots summarizing the mean mIPSC inter-event intervals (**k**) and mIPSC amplitude (**l**) in *L7-Cre* and *L7-Cre; BOD1^f/f^* mice. The insets depict the average mIPSC frequencies and amplitudes (n=20 cells from 3 mice per group; unpaired two-tailed Student’s *t* test). **m** Schematic injection of SFV-EGFP injection into the cerebellar IV/V lobe. **n** Representative images (left) and heat map (right) of PCs after SFV-EGFP injection 8 hours. **o** Quantification of intersection number in dendritic branches in *L7-Cre* and *L7-Cre; BOD1^f/f^* mice (n=3 mice per group; ***P* < 0.01; unpaired two-tailed Student’s *t* test). **p** Representative spine density in cerebellar IV/V lobe PCs in *L7-Cre* and *L7-Cre; BOD1^f/f^* mice by Lucifer yellow. **q-t** Quantification of total (**q**), mushroom (**r**), stubby (**s**) and thin (**t**) spine densities (n=3 mice per group; **P* < 0.05; ****P* < 0.001; unpaired two-tailed Student’s *t* test). The bars with error bars represent means ± s.e.m; ns, not significant.

**References**

1. Hadzipasic, M. et al. Reduced high-frequency motor neuron firing, EMG fractionation, and gait variability in awake walking ALS mice. *Proc Natl Acad Sci U S A*. **113**, E7600-E7609 (2016).
